# Exploiting ELIOT for Scaffold-Repurposing Opportunities: TRIM33 a Possible Novel E3 Ligase to Expand the Toolbox for PROTAC Design

**DOI:** 10.3390/ijms232214218

**Published:** 2022-11-17

**Authors:** Tommaso Palomba, Giusy Tassone, Carmine Vacca, Matteo Bartalucci, Aurora Valeri, Cecilia Pozzi, Simon Cross, Lydia Siragusa, Jenny Desantis

**Affiliations:** 1 Department of Chemistry, Biology and Biotechnology, University of Perugia, Via Elce di Sotto, 8, 06132 Perugia, Italy; 2 Department of Biotechnology, Chemistry and Pharmacy, University of Siena, Via Aldo Moro, 2, 53100 Siena, Italy; 3 Molecular Horizon srl, Via Montelino, 20, 06084 Bettona, Italy; 4 Kinetic Business Centre, Molecular Discovery Ltd., Theobald Street, Elstree, Borehamwood, Hertfordshire WD6 4PJ, UK

**Keywords:** PROTAC, E3 ligase, scaffold-repurposing, BRD, TRIM33, TRIM24, targeted protein degradation, molecular interaction fields

## Abstract

The field of targeted protein degradation, through the control of the ubiquitin–proteasome system (UPS), is progressing considerably; to exploit this new therapeutic modality, the proteolysis targeting chimera (PROTAC) technology was born. The opportunity to use PROTACs engaging of new E3 ligases that can hijack and control the UPS system could greatly extend the applicability of degrading molecules. To this end, here we show a potential application of the ELIOT (E3 LIgase pocketOme navigaTor) platform, previously published by this group, for a scaffold-repurposing strategy to identify new ligands for a novel E3 ligase, such as TRIM33. Starting from ELIOT, a case study of the cross-relationship using GRID Molecular Interaction Field (MIF) similarities between TRIM24 and TRIM33 binding sites was selected. Based on the assumption that similar pockets could bind similar ligands and considering that TRIM24 has 12 known co-crystalised ligands, we applied a scaffold-repurposing strategy for the identification of TRIM33 ligands exploiting the scaffold of TRIM24 ligands. We performed a deeper computational analysis to identify pocket similarities and differences, followed by docking and water analysis; selected ligands were synthesised and subsequently tested against TRIM33 via HTRF binding assay, and we obtained the first-ever X-ray crystallographic complexes of TRIM33α with three of the selected compounds.

## 1. Introduction

The field of targeted protein degradation (TPD) through the control of the ubiquitin–proteasome system (UPS) is developing considerably [1,2,3,4]. Precisely to exploit this new therapeutic modality as an alternative to classical protein inhibition, proteolysis targeting chimeras (PROTACs) technology was born, based on the use of small molecules specially designed to promote the interaction between a specific E3 ligase and the desired target protein [5,6,7,8]. PROTACs are hetero-bifunctional molecules constituted of two moieties: one able to bind a protein of interest (POI) and the other one to hijack an E3 ligase. These two moieties are joined by a linker of a variable length and type. PROTACs lead to the formation of POI–PROTAC–E3 ternary complexes, promoting forced interaction that can induce POI ubiquitination and its subsequent degradation through the cellular UPS [9,10]. PROTACs may represent a powerful tool to extend druggable space to new target types previously considered intractable or undruggable [4,11]. For example, given the complexity and complicated dynamics of the tumour environment, research for cancer treatment is constantly evolving, and recently, the TPD driven by PROTACs has attracted a lot of attention, emerging as a creative and promising approach to cancer treatment because it allows for selective degradation of oncogenic proteins that are important for cancer development [12,13,14,15]. In the UPS mechanism, the E3 ligases play a key role by dictating the specificity for the target protein to be degraded [11,13]. The opportunity to engage new E3 ligases that can hijack and control the UPS system could greatly extend the applicability of degrading molecules as a new alternative therapeutic modality [16]. Until now, most of the known PROTACs, designed for the degradation of several different protein targets, use fewer than 15 E3 ligases, and among these, mostly cereblon (CRBN) and Von Hippel–Lindau (VHL) have been exploited. The entire E3 ligase landscape is made up of more than 600 genes, and although CRBN and VHL seem to work well, their almost exclusive use may have potential disadvantages:

- the emergence of drug resistance. Resistance in cancer cells has been detected following chronic treatment with PROTACs based on VHL or CRBN [17];
- the formation of some protein–ligase complexes that do not persist long enough for ubiquitination [18,19];
- their use may be limited by their expression in human organism tissues [15].

To overcome these possible issues, the selection of the appropriate E3 ligase for a PROTAC is clearly a key step of PROTAC design [20]. Moreover, the biological function, the level of expression in the tissues, the cell localisation, and the degree of involvement of the E3 ligase in human diseases are essential factors to be considered. For example, recruiting an E3 ligase with a selective tissue expression profile presents a unique opportunity for some therapeutic applications, especially if there is a difference between the diseased and the healthy tissue [21,22]. Part of the research in the field of protein degradation aims to expand the arsenal of E3 ligases, opening new potential therapeutic possibilities [16,22,23]. In our recent work, we presented ELIOT (E3 LIgase pocketOme navigaTor, https://eliot.moldiscovery.com) [24], a platform containing the E3 ligase pocketome enabling navigation to aid the selection of new E3 ligases and their ligands for the design of PROTACs. Starting from the available human crystallographic structures in the Protein Data Bank (PDB) [25] (last update 30 November 2019), we have identified, characterised, and described all possible pockets on the surface of the E3 enzymes with the aim of unveiling new sites suitable for binding PROTACs. In ELIOT, we reported a similarity analysis among all E3 ligase pockets based on GRID molecular interaction fields (MIFs) [26,27,28,29] in order to identify similarities between binding sites [30]. The MIFs characterise pockets in terms of their three-dimensional non-bonded interaction, thus evaluating the type, strength, and direction of the interactions that a pocket is capable of making with a putative ligand. In this regard, one of the main powerful applications of ELIOT is to navigate within the pocketome, looking for similarities among the E3 ligase pockets. Considering that similar pockets could bind similar ligands, the cross-relationship analysis presented in ELIOT could provide interesting similarities between pockets for ligand-repurposing opportunities or, as explained in this work, ideas for scaffold-repurposing opportunities that can also be exploited as starting points for a structural optimisation campaign. The MIFs approach has proven to be very useful and powerful for this type of application, as demonstrated previously by our research group [31].

Here we report a case study of the cross-relationship between two binding sites of the E3 ligases tripartite motif containing 24 (TRIM24) and tripartite motif containing 33 (TRIM33). TRIM33 and TRIM24 are members of the tripartite motif (TRIM) family and they show an E3 ligase activity and are involved in the ubiquitination process of different proteins [32]. TRIM proteins are essential for a variety of functions, including cell development, tumour suppression, DNA damage repair signalling, and many others [33]. They are characterised by the presence of a conserved N-terminal tripartite motif which includes one RING domain, one or two zinc-finger domains, and an associated coiled-coil region. Some of the TRIM family members possess a C-terminal plant homeodomain (PHD) and bromodomain (BRD) modules which bind methylated lysine (KMe_n_) and acetylated lysine (KAc), respectively; TRIM24 and TRIM33 are also included among these [34]. BRDs share a conserved overall fold comprising a unique left-handed bundle consisting of four α helices linked by highly variable loop regions that form the docking site (KAc binding pocket) as their interacting recognition motif [35]. As well explained and analysed in depth by Sekirnik et al. in their recent work [36], the KAc pocket in the BRD is hydrophobic but also contains conserved structural water molecules [37] and a highly conserved asparagine residue (N140 in BRD4 [38]) that may be exploited for hydrogen bonding to KAc or to develop selective ligands. While TRIM24 has the canonical BRD, TRIM33 shows two isoforms (α and β) produced by alternative splicing, and the BRD of the canonical one (TRIM33α) differs through insertion of 17 amino acids. Due to this modification, a rearrangement of the residues occurs and moves the conserved asparagine outward from the KAc pocket, indicating that it cannot interact with KAc or putative ligands at this position. Despite this, an X-ray crystallographic structure of TRIM33α in complex with a KAc peptide is known (PDB code: 3u5o [39]), and the terminal and interactive carboxyl group of the peptide preserves its placement similarly to the carboxyl group of the known ligands and the peptides in complexes with TRIM24. Although the therapeutically relevant role is known for BRD [37,40,41], the TRIM proteins remain understudied. While for TRIM24′s BRD there are several known ligands [42,43,44], TRIM33 was still unexplored, and there were no published ligands at the start of this work despite the fact that it seems to be therapeutically interesting from a different point of view. TRIM33 is a tumour suppressor in several cancers [33,38,45,46,47], and has a key role in β-catenin degradation, acting as E3 ligase, and preventing brain tumour development and human carcinoma [34]; it has recently been reported for its negative role in the growth of prostate tumours stabilising AR and protecting it from Skp2-mediated ubiquitination and proteasomal degradation [48]. Furthermore, 276 unique TRIM33 interactors are reported in BioGRID [49]. Among these, there are several possible interesting POIs: some nuclear receptors (ERα, ERβ, ERBB), thyroid hormone responsive (THRSP), and poly(ADP-ribose) polymerase family member 1 (PARP1 in M. musculus), as well as others (the entire list of BioGRID interactors are reported in Table S1 in Supporting Information). In addition to arousing therapeutic interest for the pathologies in which it is directly involved and considering its proven E3 ligase activity, TRIM33 could be very promising to hijack the degradation of some of its already-known interactors. For all these reasons, the cross-relationship between TRIM24 and TRIM33 highlighted by ELIOT was quite interesting and a good starting point to find ligands for TRIM33 using the GRID MIFs approach. Even if TRIM24 and TRIM33 belong to the same family and show sequence similarity, the power of ELIOT consists in highlighting the similar and dissimilar 3D interactions they are able to have with putative ligands. Despite this, the main aim of this work is not to focus on the design and discovery of the best binders of TRIM33 but to show a possible application and workflow that can be followed starting from ELIOT to find ligands for a new E3 ligase. Starting from the cross-relationship analysis based on MIFs, a computational comparison of the binding sites was performed, and using the similarities and differences highlighted, a scaffold-repurposing approach was used. Docking analysis of selected compounds derived from the common scaffold of known ligands of TRIM24 was performed using TRIM33. Moreover, the key contribution of the water in the binding of the ligands in TRIM33 was highlighted using WaterFLAP (in house software for water network prediction) [50,51] to predict the placement and the relative energy of the water molecules in the protein-ligand complexes. The ligands, selected according to the scaffold-repurposing strategy, were synthesised and their binding was verified experimentally, developing and setting up an in house homogeneous time resolved fluorescence (HTRF) assay [52,53]. To reinforce the experimental results and our hypothesis of binding, crystallographic studies of TRIM33α were also carried out and the first complexes between TRIM33α and its ligands were obtained. 

## 2. Results and Discussion

### 2.1. ELIOT Application—A Workflow from MIF Comparison to Scaffold-Repurposing Strategy

When designing a new PROTAC, the choice of the E3 ligase can be a crucial step to increase the selectivity and the degradation efficiency towards the POI. The selection of a possible new E3 ligase could be: (a) tissue-related evaluation of its expression in human tissue, or (b) pathology-related considering the expression in a pathology of interest for a selective action in diseased cells, or (c) interactor-related, if interested in the degradation of one of its known interactors. However, as anticipated, the expansion of the pool of E3 ligases used for PROTACs is limited by the lack of their known ligands. In fact, when selecting novel E3 ligases of interest, it may be necessary to identify new ligands. To do that here, we present a possible workflow to exploit the GRID MIF similarity between E3 ligase pockets, providing a repurposing strategy starting from ELIOT.

**Figure 1:**
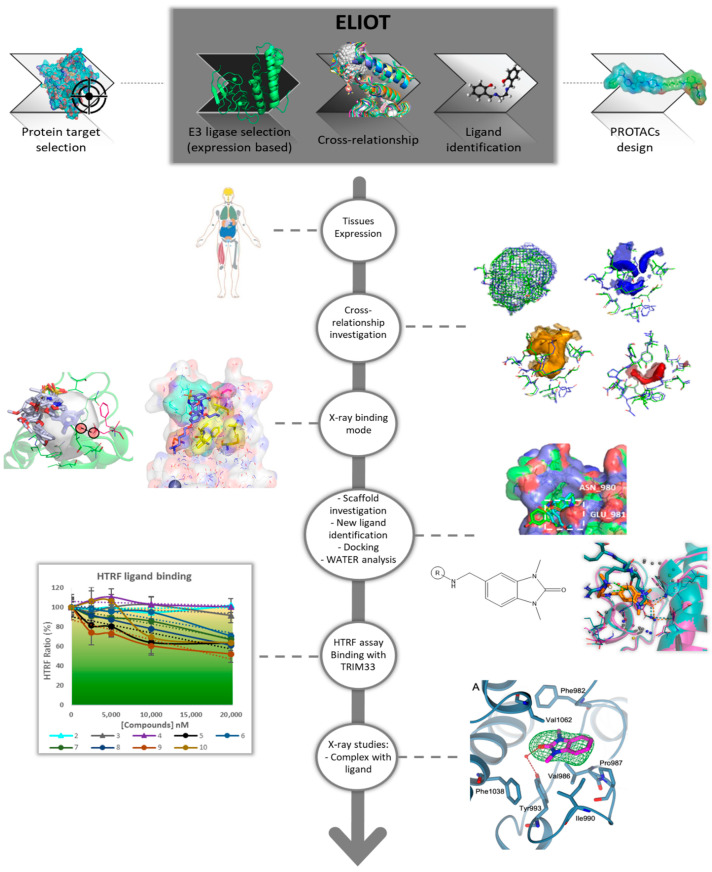
Workflow from ELIOT to identify scaffold-repurposing opportunities: a strategy to exploit the MIF-based cross-relationship between the E3 ligase pockets to identify new ligands of a novel E3 ligase.

As described above, recruiting an E3 ligase with a selective tissue expression profile presents a unique opportunity for peculiar therapeutic applications. In this regard, data from The Human Protein Atlas [54], concerning the expression of the E3 ligase in human tissues, are available in ELIOT. Once one or more interesting E3 ligases are identified, considering that most of them are not liganded, it is most likely necessary to identify new ligands. In ELIOT, the reported cross-relationship analysis compares all possible E3 ligase pockets to each other using the GRID MIFs. This can be exploited to identify similar pockets that are already liganded from the E3 ligase pocketome, and this information can be subsequently used for a ligand-repurposing or scaffold-repurposing strategy. In detail, TRIM33 was herein chosen as a case study among all the cross-relationships identified for (a) its high similarity score with a pocket of a highly liganded ligase (TRIM24), (b) the lack of any known and crystallographic ligands, (c) its promising E3 ligase activity [34], and (d) the presence of multiple known interactors and possible POIs. Once we identified TRIM33 as our E3 ligase of interest, a procedure for scaffold-repurposing application was set up involving several steps (Figure 1):

1. TRIM24–TRIM33 Cross-relationship interpretation: in-depth computational analysis to investigate the binding sites’ similarities and differences;
2. analysis of the known TRIM24 ligands and their interactions;
3. scaffold-repurposing strategy: the similarities in the binding site provide information to select an interacting and common scaffold from the known TRIM24 ligands, while the differences were exploited to identify specific substituents for TRIM33. Compounds satisfying these features were selected from the literature;
4. docking analysis to propose possible binding modes and analysis of the key role of the waters in the binding with TRIM33α;
5. HTRF assay: set up of an experimental in house procedure to evaluate the binding in TRIM33;
6. Crystallographic studies on TRIM33 to confirm the computational and experimental results.

### 2.2. TRIM24 and TRIM33 Pockets Cross-Relationship

**Figure 2:**
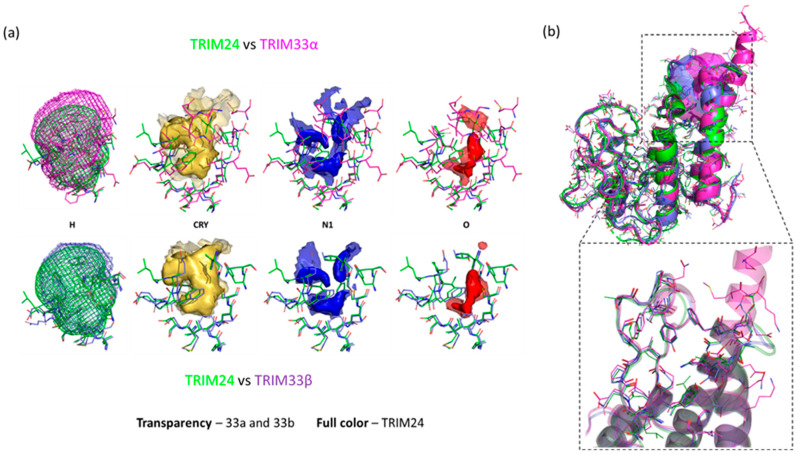
(**a**) Representation of the cross-relationship results between TRIM24 (residues in green and MIFs in transparency), TRIM33α (residues in magenta and MIFs in full colour), and TRIM33β (residues in purple and MIFs in full colour) pocket. The overlapped GRID MIFs are contoured and coloured in the following manner: H (shape) at 0.10 kcal/mol and coloured according to the relative residues, CRY (lipophilic features) in yellow at −0.6 kcal/mol, N1 (donor features) in blue at −4.5 kcal/mol and O (acceptor features) in red at −3.5 kcal/mol. (**b**) Aligned structures and pockets (TRIM24 PDB code: 4yat [42] in green, TRIM33α PDB code: 3u5o [39] in magenta, and TRIM33β PDB code: 5mr8 [36] in violet) pre-processed with BioGPS [30] with a zoomed view into the KAc binding region.

The cross-relationship analysis reported in ELIOT showed a high similarity score between TRIM24 and TRIM33 (both isoform α and β) binding sites. The similarity score is obtained by exclusively evaluating the MIF similarities (see Section 3 for details). The score ranges from 0.0 for pockets with completely different MIFs, to 1.0 for pockets with identical MIFs. In Figure 2a the overlap of the MIFs is reported. A high degree of similarity, both considering the lipophilic/hydrophobic surface (CRY MIF) and the polar surfaces (N1 and O MIFs) was detected. The comparison of the binding site of TRIM24 and TRIM33β returned a similarity score of 0.89, while the comparison between TRIM24 and TRIM33α was 0.78. This difference is due to the insertion of 17 residues on the BRD of TRIM33α, which make the binding site not canonical: it is bigger and extended upward. The MIF-based comparison, as explained, detects similar pockets simulating their possible interaction with a putative ligand. This pocket-based approach is a valid alternative to the more common sequence-based approach that was used by Sekirnik et al. [36]. They presented a characterisation of the TRIM subfamily, focusing on the investigation of the TRIM33 PHD-BRD (particularly the β isoform) from the point of view of the sequence. They reported the structural similarity based on the entire amino acid sequence between TRIM24 and TRIM33:TRIM24 PHD-BRD had 62.0% identity and 75.5% similarity with TRIM33α and 67.8% identity and 82.5% similarity with TRIM33β (Figure 2b for the entire structural superimposition).

If compared to the sequence-based approach, the advantage of using MIFs is to obtain direct information about possible interactions of the pockets. In some cases, comparing binding pockets rather than the entire protein structure may be advantageous: indeed, similar proteins, in terms of sequence, can differ for a few key residues responsible for the interaction of potential binders. On the other hand, even protein regions with different sequences could be similar in terms of the type of interactions that they can establish with a putative ligand. For these reasons, even if TRIM33 and TRIM24 exhibit quite a high sequence similarity, only a deep 3D analysis, carried out with MIFs, can reveal where and how they are actually similar and dissimilar in terms of how a ligand may interact.

### 2.3. Known X-ray Crystallographic TRIM24 Ligands 

**Figure 3:**
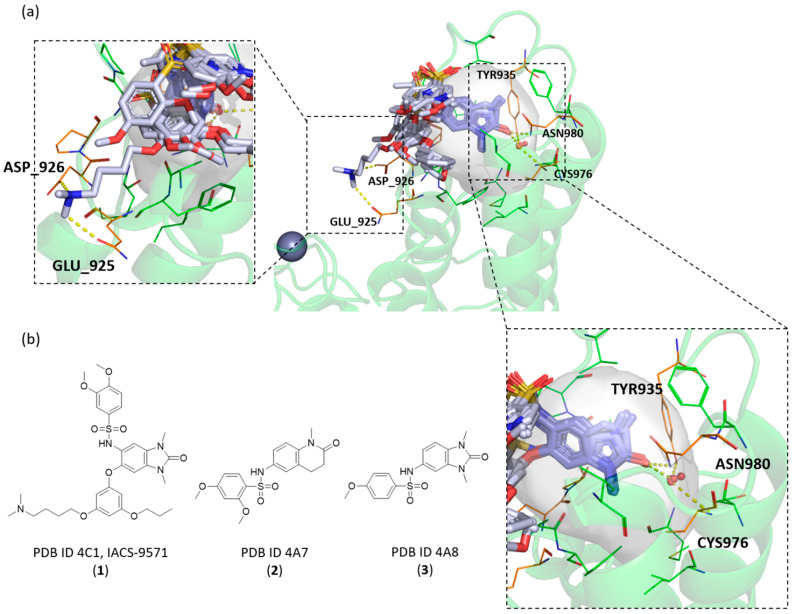
(**a**) Structure of TRIM24 (PDB code: 4yat [42]) and its known X-ray ligands coming from other TRIM24 aligned structures (PDB codes: 4yc9, 4yad, 4ybm, 4ybs, 4ybt, 4zql, and 4yax) [42]. A common binding mode of the ligands is shown, and the polar interactions with the conserved asparagine (ASN980) and the network of the conserved water molecule are highlighted. An additional interaction with the backbone of the aspartic acid (ASP926) is reported. (**b**) Chemical structures of three TRIM24 ligands (compounds **1**–**3**).

For TRIM24, eighteen X-ray crystallographic structures were collected in ELIOT (from PDB as of November 2019). Some of them presented ligands or peptides inside the KAc binding pocket, while others were in the ligand free state. In Figure 3a, a representative structure of TRIM24 (PDB code: 4yat [42]) and some of its known X-ray ligands are reported. The surface area in grey represents the binding site identified during the E3 pocketome construction in ELIOT. The reported known X-ray compounds come from different structures of TRIM24 (See Table S2 in Supporting Information for the considered PDB codes) aligned onto 4yat to show their interactions and their common binding mode. They share a similar core which takes advantage of a specific interaction with the deep and conserved asparagine residue in the pocket (ASN980 in TRIM24). In addition, in the bottom of the pocket, in almost all the structures investigated, a conserved water molecule is reported. This contributes to the stability of the complexes by forming a water network with other water molecules and interacting directly with THR953 and CYS976. Considering its position and the distance from the ligands, it may also make direct hydrogen-bond interactions with the carbonyl group of the ligands. 

**Figure 4:**
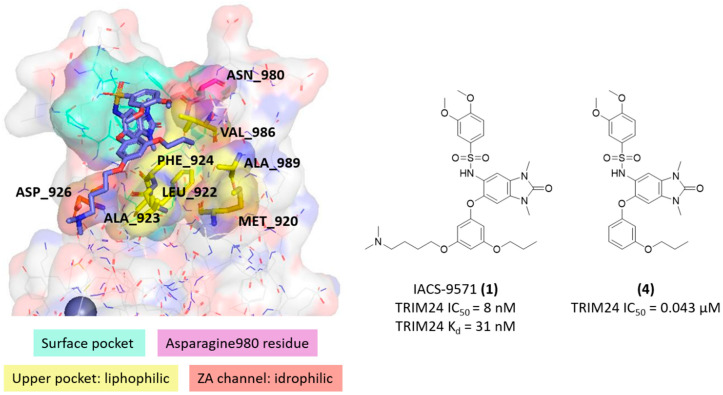
Analysis of the complex between TRIM24 and IACS-9571 (PDB code: 4yc1 [42]). The main contact regions and relative residues are highlighted. The structure of the IACS-5791 (**1**) and its less functionalised analogue (**4**) with their literature activity values against TRIM24 are reported [37].

In Figure 3b, for example, three known ligands of TRIM24 are reported: the IACS-9571 [42] (**1**) is the most active among the several TRIM24 inhibitors, while compound **2** (PDB ID 4A7) [42] and **3** (PDB ID 4A8) [42] were commercially available. As explained by Palmer et al., to improve the cellular potencies of TRIM24 inhibitors, they designed a strategic bidirectional aryl group ether in order to exploit additional lipophilic interactions in the upper pockets and mostly the salt bridge of a dimethylamino group with the ASP926 residue in the ZA channel. In addition to the main common interaction with the ASN980 in the bottom of the pockets, just the presence of this amino terminal chain seems to be the key to improve the potency, as explained by the differences in IC_50_ between the IACS-9571 and its analogue **4** (7e in Palmer et al.’s work [42]). In Figure 4, the analysis of the IACS-9571 binding mode is reported (PDB code: 4yc9 [42]). In detail, the surface pocket (in aqua green) and three main contact regions are highlighted: the ASN980 in magenta, the lipophilic upper pocket in yellow, and the hydrophilic ZA channel in red. Analogues regions were highlighted also in TRIM33α and TRIM33β (See Figure S1 in Supporting Information). 

### 2.4. Similarities and Differences Highlighted Using the MIFs Approach 

**Figure 5:**
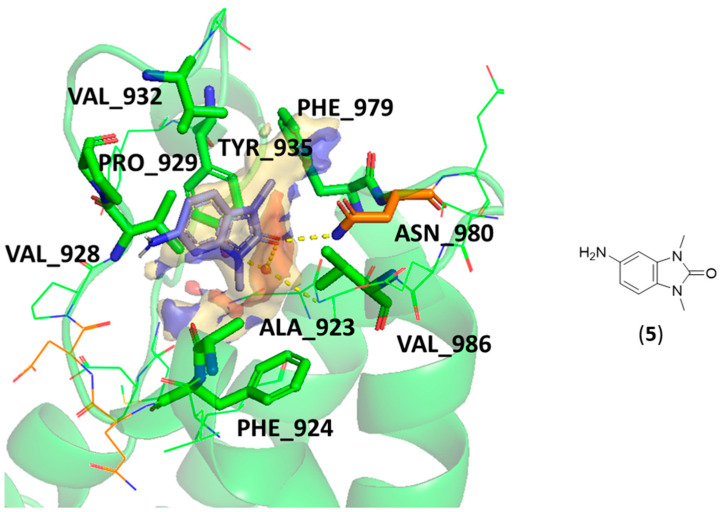
Pharmacophoric scaffold structure (exemplified by compound **5**) of the X-ray TRIM24 ligands and its binding mode from a MIFs point of view. The GRID MIFs of the TRIM24 pocket (PDB cod: 4yat [42]—ligand 4A8 was manually edited) are contoured and coloured in the following manner: H (shape) at 0.10 kcal/mol and coloured according to the relative residues; CRY (lipophilic features) in yellow at −0.6 kcal/mol, N1 (donor features) in blue at −4.5 kcal/mol, and O (acceptor features) in red at −3.5 kcal/mol. The main interacting residues are represented as sticks.

Considering the structure of the TRIM24 ligands, a common pharmacophoric scaffold was identified (exemplified by compound **5**). In Figure 5, its binding mode in TRIM24 was considered. The pocket GRID MIFs represent the binding pocket interactions, and it is evident how the ligand matches quite well the predicted interacting regions of the site. In particular, the carbonyl group overlies the acceptor MIF close to the donor group of the ASN980, and the aromatic ring overlaps perfectly the CRY MIF induced by a network of several lipophilic residues: ALA923, PHE924, VAL928, PRO929, VAL932, TYR935, PHE979, ASN980, and VAL986. 

**Figure 6:**
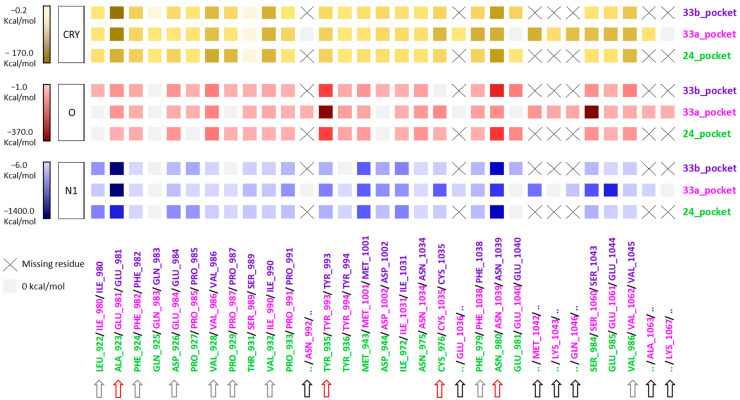
Heatmap accounting for the energy contribution of each residue that defines the KAc binding site of TRIM24, TRIM33α, and TRIM33β to the GRID MIFs CRY, N1, and O. The colour gradation from a lighter tone to a darker tone indicates a greater energetic contribution to the MIFs. If a certain residue does not contribute to the MIF, it is reported as grey. Moreover, the arrows at the bottom highlighted some interesting comparisons. Black arrows: missing residue in TRIM24 and TRIM33 β (also indicated with the symbol “X” in correspondence with the MIFs). Grey arrows: analogies for the hydrophobic interactions. Red arrows: main differences in terms of the type of residues and/or energetic contribution.

These considerations led to a deeper computational analysis in order to compare the amino acid residues that constitute the binding sites of TRIM24 and TRIM33 (both isoforms), considering their energetic contribution to the MIFs of the pockets. This type of analysis aims to identify the similarities and differences, in energetic terms, between the binding sites and consequently to aid the process of identification of TRIM33 ligands (See Section 2.5). First, we tried to understand how each residue contributes to the global interaction map of the binding sites (mentioned energy values are reported in Tables S3–S5 in Supporting Information). In the heat map reported in Figure 6, the colour gradation from a lighter tone to a darker tone indicates a greater energetic contribution of a specific residue to the MIFs. If a certain residue does not contribute to the MIF, it is reported as grey. Moreover, three types of arrows at the bottom highlight some interesting comparisons. (a) The black arrows show which residues define the TRIM33α pocket but are missing in TRIM24 and TRIM33β (also indicated with the symbol “X” in correspondence of the MIFs). (b) The grey arrows pick out conserved hydrophobic interactions (CRY GRID MIF) among the three sites with TRIM24 ligands (LEU922, PHE924, ASP926, VAL928, PRO929, VAL932, PHE979, and VAL986). Several of these residues are also conserved in TRIM33, and although some residues differ (ILE980 instead of LEU922, GLU984 instead of ASP926, and ILE990 instead of VAL932) their type of interaction and energetic contribution to the CRY MIF is the same. (c) Red arrows show the main differences in terms of the type of residues and/or energetic contribution. As shown previously, while the key asparagine residue is conserved both in TRIM24 (ASN980) and in TRIM33β (ASN1039), in TRIM33α, this residue is oriented out of the pocket, and this is also evident in energetic terms. In fact, its contribution to the N1 and CRY MIFs is the same (acceptor and lipophilic behaviour, respectively), while the contribution to the O MIF is weaker than in TRIM24 and TRIM33β. The O MIF indicates the ability of the residue to donate a proton in a hydrogen bonding interaction, and its lower value could be explained considering that the different orientation of the asparagine moves the donor group outside, resulting in no obvious interacting donor group. To compensate for the lower contribution of asparagine to the O MIF, in TRIM33α the contribution of TYR993 (TYR935 in TRIM24) and SER1060 (SER984 in TRIM24) is greater. At the same time, the energy contribution of the CYS1035 (instead CYS976) to the N1 MIF is also greater if compared to TRIM24 and TRIM33β. These differences suggest that although the interaction with the key residue of asparagine in TRIM33α is missing, the scaffold of the TRIM24 ligands could still bind TRIM33α, maintaining the same hydrophobic interactions and exploiting the greater contribution of TYR993, SER1060, and CYS976 residues to the polar MIFs in the bottom of the pocket. Another important difference is the glutamic acid residue (GLU981) at the entrance of the pocket in TRIM33 isoforms instead of an alanine residue (ALA923) in TRIM24. The GLU981 gives a more polar character to the binding region, and this is also shown in the heat map. In fact, in TRIM33 isoforms, the energetic contribution of the GLU981 to the N1 MIF is greater than the energetic contribution of the ALA923 in TRIM24. At the same time, the energetic contribution of GLU981 to the CRY MIF also increased. Furthermore, this residue occupies a greater volume close to the entrance of the pocket and probably regulates its access. Briefly, the main similarities between the three binding sites are due to the lipophilic residues that occupy the central region of the pockets (LEU922, PHE924, ASP926, VAL928, PRO929, VAL932, PHE979, and VAL986 in TRIM24) while the main differences are due to the presence of the glutamic acid residue (GLU981) in TRIM33, which replaces an alanine residue (ALA923) in TRIM24 and the different orientation of the key asparagine residue (ASN980 in TRIM24 and ASN1039 in TRIM33β conserved the canonical conformation, while in TRIM33α it is facing outwards resulting in a significant decrease in the O MIF).

### 2.5. Scaffold-Repurposing Strategy: Searching for TRIM33 Ligands

**Figure 7:**
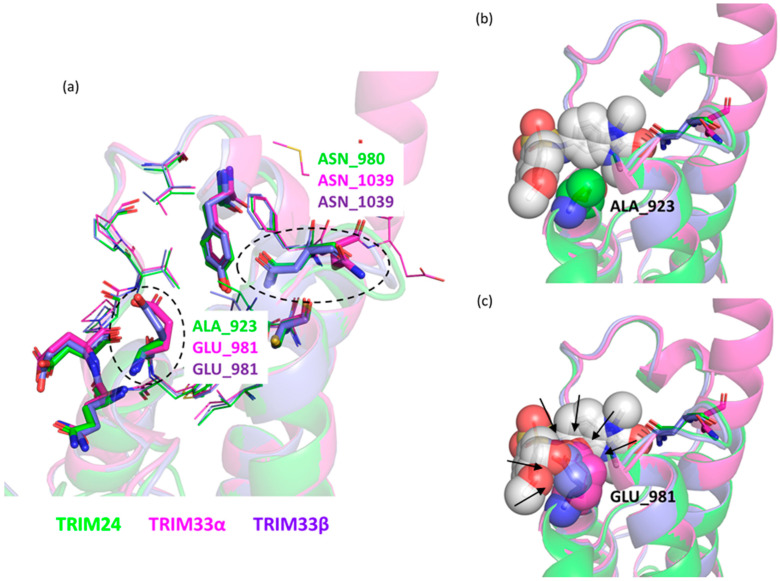
View of the KAc binding region of the aligned X-ray crystal structures (TRIM24 PDB code 4yat [42] in green, TRIM33α PDB code 3u5o [39] in magenta, and TRIM33β PDB code 5mr8 [36] in violet). (**a**) The main interacting polar residues are represented as sticks. Differences are highlighted: ALA923/GLU981 and ASN980/ASN1039. (**b**) Ligand 3 (PDB id 4A8) in its X-ray pose is represented as spheres coloured by element with its carbon atoms in light grey and the ALA923 residue in TRIM24 also. (**c**) Ligand **3** (PDB id 4A8) in its X-ray pose in TRIM24 is represented as spheres (coloured by element, with its carbon atoms in light grey) aligned to TRIM33s and the GLU981 residue in TRIM33s is shown as spheres to highlight possible clashes (indicated by arrows) between ligand and protein.

The comparison of the energetic contribution to the MIFs has allowed us to identify not only the main similarities but also the presence of a few significant differences (Figure 7). For this reason, a scaffold-repurposing strategy was preferred over that of ligand-repurposing.

The conserved lipophilic character and polar spot in the bottom of the pocket of TRIM33 allowed us to preserve the common scaffold (**5**) of the TRIM24 ligands also for TRIM33. However, the presence of the GLU981 in TRIM33 may limit the access of the known compounds of TRIM24. In Figure 7, the aligned structures of TRIM24, TRIM33α, and TRIM33β are reported and the possible clashes between the TRIM24 ligand **3** (PDB ID 4A8) and the GLU981 residue in TRIM33 (both isoforms) are highlighted (Figure 7c). 

The structural differences highlighted with the energy treatment of MIFs suggested structural modifications of the TRIM24 ligands in order to exploit the selective polar interaction with GLU981 and replicate the additional interaction that increased the potency of the IACS-9571 with the ASP926 residue (GLU984 in TRIM33s). 

**Figure 8:**
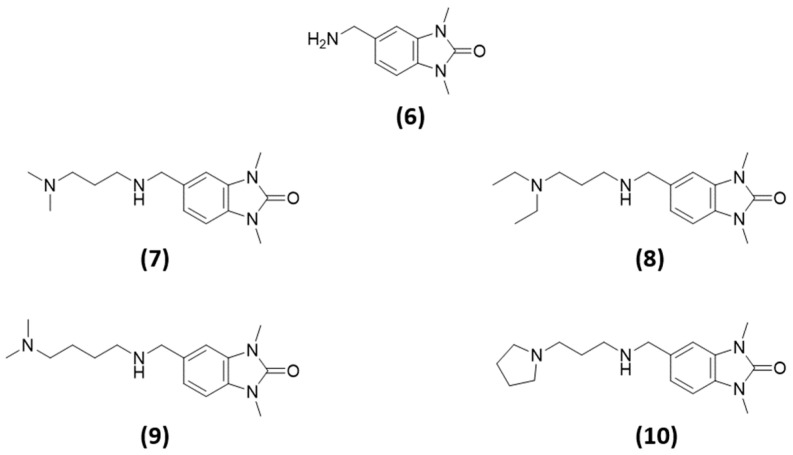
Chemical structures of the putative ligands of TRIM33, selected according to the scaffold-repurposing strategy.

Looking for ligands with these characteristics, an interesting series emerged [55]. These compounds have the same scaffold of TRIM24 ligand (scaffold **5**) except an additional methylene group as spacing between the aromatic ring of the core and the amino group (scaffold **6**). This should push the amino group closer to GLU981, allowing for better interaction. Moreover, these compounds also have an alkyl amino terminal side chain of 3 or 4 methylene units attached to the amino group. This architecture should allow the ligands to maintain all main interactions of the TRIM24 scaffold (**5**) and make additional interactions with GLU981 and GLU984. In Figure 8, the structures of the selecting compounds are reported.

### 2.6. Docking Analysis for TRIM33α and TRIM33β—The Key Role of a Water Molecule 

The docking analysis was performed (a) to understand the role of the differences showed with the MIFs comparison between TRIM24 and TRIM33 pockets, (b) to highlight the main differences in the binding mode of the compounds induced by the presence of the GLU981 in TRIM33 (instead of ALA923 in TRIM24), (c) to rationalise the hydrogen bonding in TRIM33α considering the different orientation of the asparagine residue, and (d) to evaluate the possible key role of the conserved water molecule in the bottom of TRIM33α binding site.

#### 2.6.1. Water Consideration

As previously discussed, the analysis of the X-ray structures of TRIM24 co-crystalised with its ligands (Figure 3) highlighted the presence of a structural water in the bottom of the KAc pocket. Generally, the presence of this water is a peculiarity of the KAc binding site of the BRDs and it is also conserved in one of the TRIM33α X-ray structures (PDB code: 3u5n [39]). This water makes structural network interactions with TYR935 and CYS976 in TRIM24 and it may also interact with the ligand. In TRIM33α, that water could have a key role interacting with the carbonyl group and replacing the interaction that in TRIM24 and TRIM33β is made with the ASN980 (See Figure S2 in Supporting information). Consequently, the conserved X-ray water was used as part of the receptor to bias the docking in TRIM33α. 

#### 2.6.2. Docking Analysis

The docking analysis involved three known ligands of TRIM24 (**2**, **3**, and **4**), the most reduced fragments **5** and **6,** and compounds **7**, **8**, **9**, and **10**.

**Figure 9:**
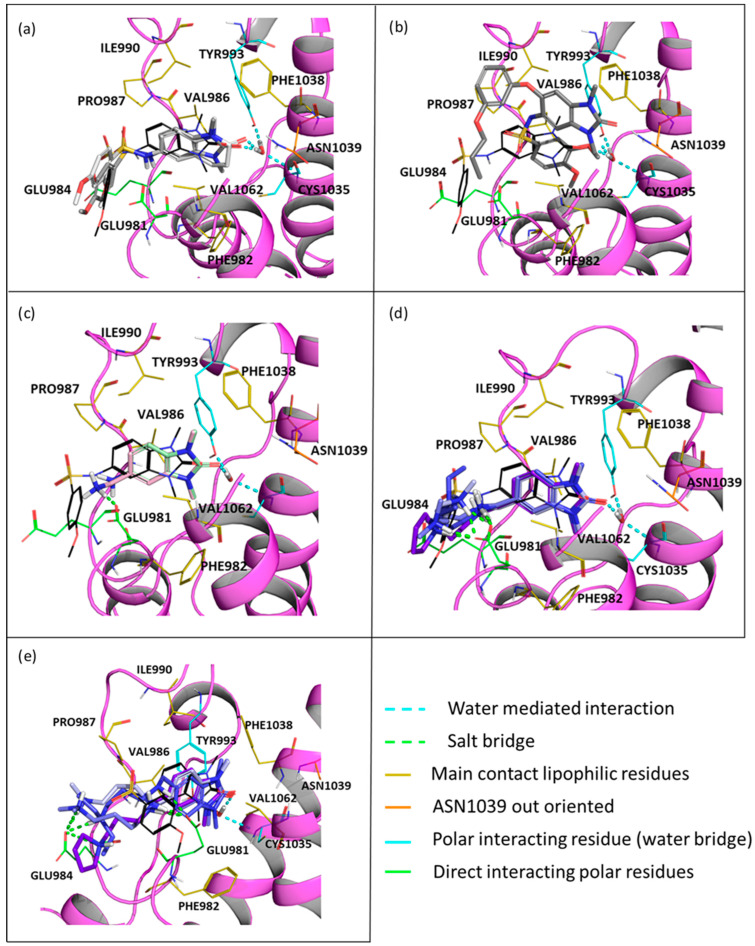
The obtained docking poses in BRD of TRIM33α (PDB code: 3u5o [39], represented in magenta) using GLIDE are reported. Reference X-ray ligand **4** (PDB ID 4A8) for its binding mode (represented as black wire). The main interacting pocket residues are highlighted: lipophilic residue as yellow wires, ASN1039 as orange wires, TYR993 and CYS1035 as cyan wires and GLU981 and GLU984 as green wires. Moreover, the water-mediated interaction as represented in cyan and the salt bridge interactions in green. (**a**) Representation of compounds **2** (as light grey sticks) and **3** (as grey sticks). (**b**) Representation of compound **4** (as dark grey sticks). (**c**) Representation of scaffold **5** (as pale green sticks) and **6** (as light pink sticks). (**d**) Representation of compound **7** (as light blue sticks), **8** (as tv blue sticks), **9** (as slate sticks), and **10** (as purple sticks) in their first possible conformation. (**e**) Representation of compound **7** (as light blue sticks), **8** (as tv blue sticks), **9** (as slate sticks), and **10** (as purple sticks) in their second possible conformation.

- In TRIM33α, the obtained poses for all the tested compounds replicate the reference binding mode of the X-ray ligand **3** (represented as the black wire in Figure 9).
  - The presence of the glutamic acid residue (GLU981) should not allow the TRIM24 compounds to fit properly in the pocket. Despite this, for compounds **2** and **3,** good poses in TRIM33α were found (Figure 9a) contrary to what was previously assumed, while for compound **4,** no plausible poses were obtained (Figure 9b).
  - Both scaffold **5** and **6** fit properly in the pocket, but scaffold **6** can also exploit the salt bridge with GLU981 (Figure 9c).
  - All the specific TRIM33 ligands (**7**, **8**, **9**, and **10**) fit very well in the pocket (Figure 9d,e).

It is evident how in TRIM33α, the water molecule plays a key role in binding by replacing the interaction of the ligands with the asparagine residue which is oriented outside. The same docking analysis without considering the water but with the same parameters returned completely wrong poses (see Figure S3 in Supporting Information for details). The comparison between scaffolds **5** and **6** (Figure 9c) highlights the role of the added methylene group between the benzimidazolone scaffold and the amino group. By inserting the spacer, the amino group becomes close to the GLU981 with which it forms a salt bridge interaction. Furthermore, ligands **7**, **8**, **9**, and **10** also exploit another salt bridge anchoring between the terminal amino group of the added chain and the same GLU981 at the entrance of the pocket (Figure 9d) or, due to the high mobility of the aliphatic chain, ligands could assume a conformation with the chain stretched and forming a salt bridge with the GLU984 of the backbone (Figure 9e). Despite that, all the obtained poses seem qualitatively possible (except for compound **4**), the docking score for compounds **2** and **3** (coming from TRIM24) is lower than the docking score of the other TRIM33 compounds, confirming the hypothesis of a lack of bonding or lower affinity towards TRIM33. Some considerations in this regard were made:

- The salt bridge with GLU981 is missing because the amide group can not establish it. Furthermore, even a possible hydrogen bond interaction between the amide and the GLU981 should not occur as at pH 7.4, the amide of the most abundant protomer (66.9%—predicted pK_a_ = 7.19 [56]) of compounds **2** and **3** is deprotonated. Additionally, the reduced mobility due to the rigidity of the sulfonamide does not allow the correct directionality of the amide group towards the glutamic acid.
- As discussed above, after aligning the crystallographic pose of the TRIM24 ligand onto TRIM33α, it is evident how the glutamic acid residue in TRIM33α prevents the access of the ligand to the binding site. The dimethoxybenzenesulfonamide group in its TRIM24 conformation clashes with TRIM33. For this reason, during the docking process, ligands should adapt to the presence of the glutamic acid residue, but their rigidity does not allow them to assume the optimal conformation.
- The absence of an amino terminal aliphatic chain does not allow them to establish additional interactions with the backbone.

- In TRIM33β, the obtained poses are similar to the ones obtained in TRIM33α, except for the main interaction of the core. In this case, the asparagine residue is well-oriented and it can interact directly with the ligand (as in TRIM24), and the conserved water makes a water mediated interaction with the TYR993 and CYS1035 residues (See Figure S4 in Supporting Information). The observations and differences in the binding mode and the docking score of the compounds are the same as those made previously for TRIM33α.

#### 2.6.3. WaterFLAP Analysis Confirms the Key Role of the Water in TRIM33α Bond

WaterFLAP [50,51] is an in house software for the prediction of the placement and energy of the water molecules in proteins and protein-ligand complexes. This evaluates the impact of the ligand on the water network of the binding site and the effect and contribution of the waters toward binding (See Section 3 for details). The WaterFLAP analysis, including the X-ray pose of compound **3** for TRIM24 and the docking pose of **9** in TRIM33α as part of the receptor, confirms the structural role of the water in TRIM24 and its key role in TRIM33α to conserve the binding mode of the ligands.

**Figure 10:**
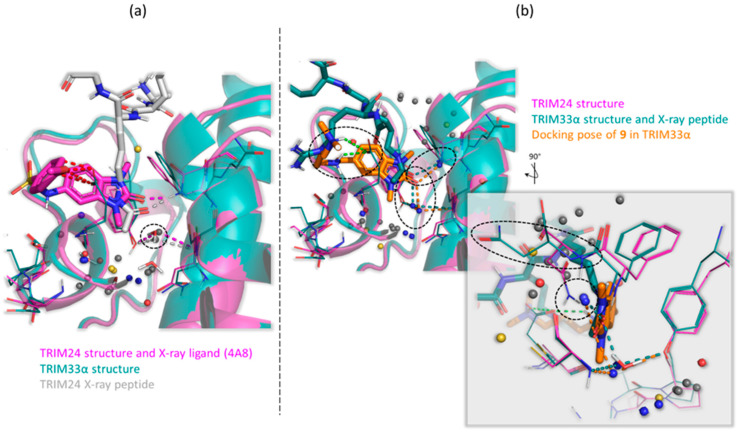
Prediction of the water molecules in the KAc binding site of TRIM24 and TRIM33α. The aligned structures of TRIM24 (PDB code: 4yat [42] in magenta) and TRIM33α (PDB code: 3u5o [39] in teal) are reported. The coloured spheres represent the predicted water molecules using WaterFLAP. The colour is related to their predicted free energy value (∆G): RED for “Very unhappy” water (∆G ≥ 3.0 kcal/mol); YELLOW for “Unhappy” water (1.5 kcal/mol ≤ ∆G < 3.0 kcal/mol); GREY for “Solvent-like” water (−2.0 kcal/mol ≤ ∆G < 1.5 kcal/mol); BLUE for “Happy” water (∆G < −2.0 kcal). (**a**) TRIM24 X-ray crystallographic compound **3** (PDB ID 4A8 in magenta) and TRIM24 X-ray crystallographic peptide (PDB code: 3o35 [57] in light grey). The X-ray water molecule is reported as sticks. (**b**) Compound **9** (in orange) docked into TRIM33α and TRIM33α X-ray crystallographic peptide (PDB code: 3u5o [39] in teal). The X-ray water molecule used as a constraint for the docking is reported as sticks.

In Figure 10, the aligned structures of TRIM24 and TRIM33α are reported. The coloured spheres represent the predicted water molecules, and their colour is related to their relative free energy value (coming from a contribution of entropic and enthalpic terms). Figure 10a shows the interactions of compound **3** and peptide with ASN980 of TRIM24. Using the complexes of TRIM24 both with the ligand and the peptide, WaterFLAP reproduces the placement of the conserved water, and in both cases, waters are classified as solvent-like (the energy is close to 0 kcal/mol). This implies that the conserved water molecule is not involved in any strong interactions with the ligands. Figure 10b shows the interactions of the docked compound **9** and the X-ray peptide in TRIM33α. The X-ray water used to bias the docking is also included. The interaction of the ligand with GLU981 and with the water molecules are shown. In this case, also using the complexes of TRIM33α both with the docked ligand and the peptide, WaterFLAP well predicted the placement of the conserved water. Differently from TRIM24, in TRIM33α, its energy is lower than −2 kcal/mol and it is classified as happy; in other words, it has more characteristics of a structural water. The energy gain means that this water is likely involved in interactions both with CYS and TYR (as in TRIM24) but also with the ligand/peptide. Additionally, the front picture of the complexes with TRIM33α also shows an additional blue happy water that can interact with the ligand/peptide and which WaterFLAP predicts to fill the empty spot left by the different orientation of the ASN1039. Moreover, a slight difference in the direction of the carbonyl towards the bottom of the pockets, compared to complexes with TRIM24, in which the carbonyl group is facing to the asparagine residue, is shown. This study confirms the key role of the water in the binding of TRIM33α. As support of the interactions of the added amino terminal chain with GL981 and GLU984, the scaffold exploits the conserved water molecule and probably also another predicted water molecule which replaces exactly the position occupied by the asparagine residue in TRIM24.

### 2.7. HTRF Assay

To validate the scaffold-repurposing strategy followed, an HTRF assay to detect the binding in TRIM33 was set up in house. Compounds **2** and **3** were purchased, while **4**, **5**, **6**, **7**, **8**, **9**, and **10** were synthesised in house (See Supporting Information for details). 

#### 2.7.1. Identification of a Peptide with the Highest Affinity towards the BRD of TRIM24, TRIM33α, and TRIM33β

Three commercial peptides were tested: biotinylated histone H_3_K_23_Ac peptide, biotinylated histone H_4_K_5,8,12,16_Ac peptide, and biotinylated histone H_3_K_14_Ac peptide. Their affinity was measured against TRIM24, TRIM33α, TRIM33β, and TRIM28 also. This latter was used as a negative control. In fact, while TRIM24 and TRIM33β have the canonical KAc binding pocket of most BRDs and TRIM33α, despite its insertion of 17 residues and consequent rearrangement of the asparagine residue binds H3 peptides, TRIM28 does not present the canonical BRD domain. In particular, the conserved asparagine is replaced by a threonine residue and due to the different rearrangement of the residues and the different conformation in the KAc binding region of TRIM28 no binding pocket was identified and reported in ELIOT (see Figure S5 Supporting Information).

**Figure 11:**
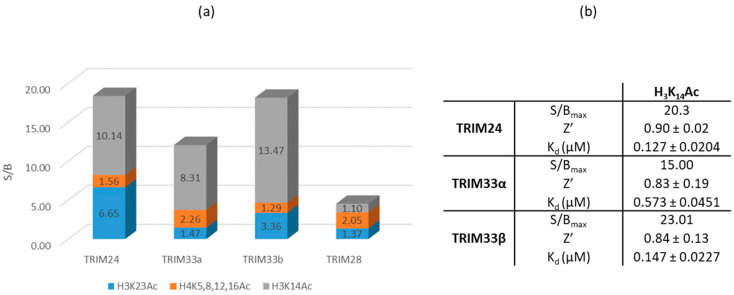
(**a**) Preliminary HTRF assay to evaluate the affinity of 3 different peptides against TRIM24, TRIM33α, TRIM33β, and TRIM28 (as negative control). The value reported refers to the S/B ratio detected using histone peptide at a concentration of 3 µM. (**b**) S/B ratio, Z’ and K_d_ values were calculated performing titration curves of H_3_K_14_Ac with TRIM24, TRIM33α, and TRIM33β. Values quoted are the mean of triplicate data ± standard error of the mean.

Results of the HTRF assay are reported as the S/B (signal/background) ratio (see Section 3 for the procedure). In Figure 11a, the S/B signal of the tested peptide against TRIM24, TRIM33 (both isoforms), and TRIM28 is reported. A ratio value close to 1 means that there is not any bond between the protein and the peptide.

The experimental results displayed in the chart show the common behaviour towards TRIM24, TRIM33α, and TRIM33β of the peptides despite the different orientations of the asparagine residue in TRIM33α. Their BRD binds with higher affinity to the H3 peptide. In particular, the greatest affinity was obtained with H3K14Ac. Moreover, the HTRF assay confirmed how none of the peptides binds the TRIM28 protein (S/B close to 1) as evidence of the absence of a canonical KAc binding site in its BRD. 

In order to better characterise the binding between the histone H_3_K_14_Ac and the proteins, an HTRF assay with three titration curves were performed, one for each TRIM (see Section 3 for more details). In this way, by varying the concentration of the peptide, it was possible to calculate the K_d_ (dissociation constant) of each protein/peptide system (relative curves are reported in Figure S6 in Supporting Information) and identify the optimal concentration to be used in subsequent competition assays.

#### 2.7.2. HTRF Competition Assay to Identify TRIM33 Ligands

HTRF competition assays were performed to test the binding of compounds. In the presence of a ligand, this will compete with the biotinylated histone for binding with the protein. If the ligand binds the protein, it causes a decrease in the energy transferred (between the protein and the peptide), resulting in a decrease in fluorescence and then in the S/B ratio. This decrease will be proportional to the strength of the bond between the compound and the protein. Due to the high sensitivity of the assay and the small volume of solution used (final volume 20 µL), a significant signal decrease was considered only if greater than 20%.

**Figure 12:**
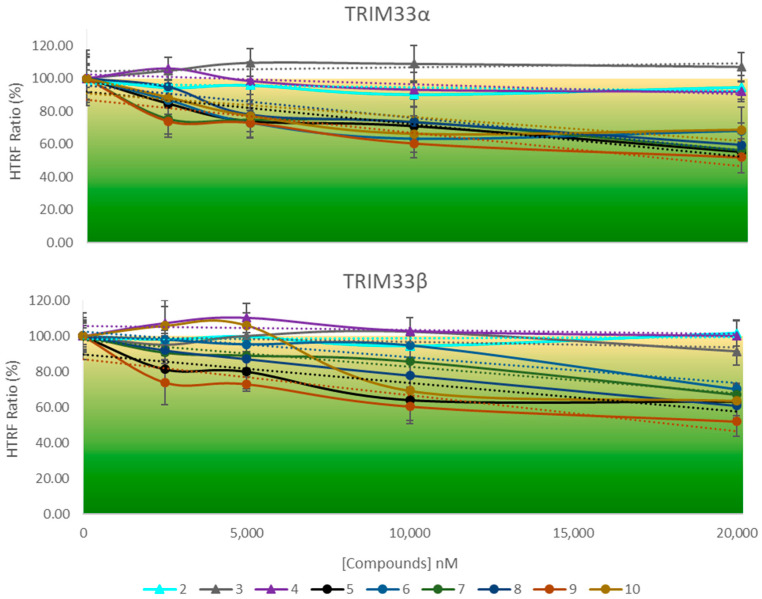
Dose-response HTRF competition assay against TRIM33α and TRIM33β. Curves related to compounds **2**, **3**, **4**, **5**, **6**, **7**, **8**, **9**, and **10** are reported with different colours. The HTRF ratio at different concentrations of the compounds and using a fixed peptide concentration (K_d_) was detected. Values are the mean of three independent experiments ± standard error of the mean.

The graphs below (Figure 12) show the curve related to the results of the performed HTRF assay in TRIM33α and TRIM33β with compounds **2**, **3**, **4**, **5**, **6**, **7**, **8**, **9**, and **10**.

Results obtained confirm that the selected ligands, according with the scaffold-repurposing strategy, bind TRIM33 because there is a progressive lowering in the signal when the concentration of the compound increases. In contrast, TRIM24 ligands **2**, **3**, and **4** did not bind TRIM33 (both isoforms). These differences can be observed at 200 µM where there are two distinct groups of compounds: binders and not binders. All TRIM33 ligands (binders) seem to have a similar affinity for the TRIM33 isoforms. They induce a decrease of the signal in the range of 30–40% (the detected signals are in the range of 70–60%). Among these, compound **9** (brown curve) is slightly the better one (decrease of the signal bigger than 40%). Although **7**, **8**, **9** and **10** bind TRIM33, they should not have an optimal potency since high micromolar concentration must be reached in order to appreciate significant signal variations. For this reason, it was not possible to calculate an IC_50_ via HTRF. However, the different responses appreciated in the HTRF are qualitative enough to distinguish binders from non-binders of TRIM33.

#### 2.7.3. Correlation between Docking and HTRF Assay

**Figure 13:**
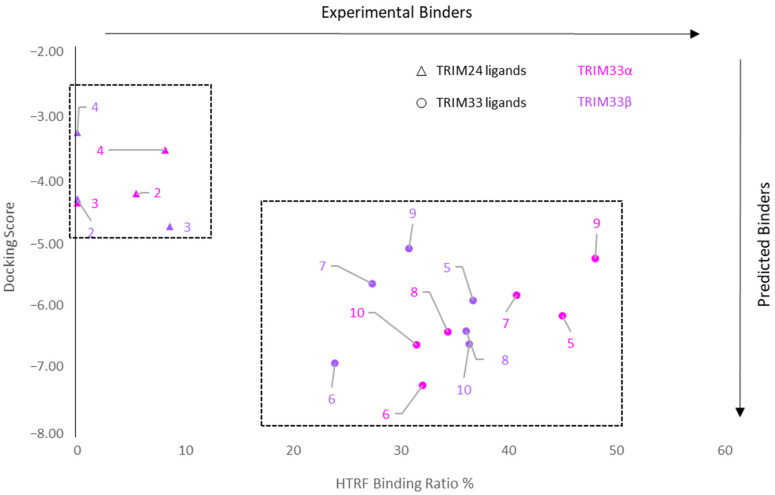
Correlation plot between the predicted binders (docking score) and experimental binders (detected HTRF signal). TRIM24 known ligands show as a triangle, and TRIM33 ligands show as round.

In the plot below (Figure 13), the correlation between the docking scores (Y-axis) and the decrease of the HTRF signal (X-axis) is reported. In particular, considering the quantitative aspect of the docking, a good consistency between the predicted and experimental results is shown. The rectangular boxes highlighted two main “clusters”. The one on the top left highlights the TRIM24 ligands, which do not bind TRIM33α and TRIM33β (HTRF Binding Ratio < 10% and lower docking score), while the box in the centre highlights the TRIM33 ligands (HTRF Binding Ratio > 25% and better docking score).

### 2.8. Crystallography

To confirm the followed scaffold-repurposing strategy and to validate the experimental data obtained, crystallographic studies were also performed for compounds **8**, **9**, and **10,** which emerged as the most potent TRIM33 binders as well as showing the best affinities in the docking analysis.

**Figure 14:**
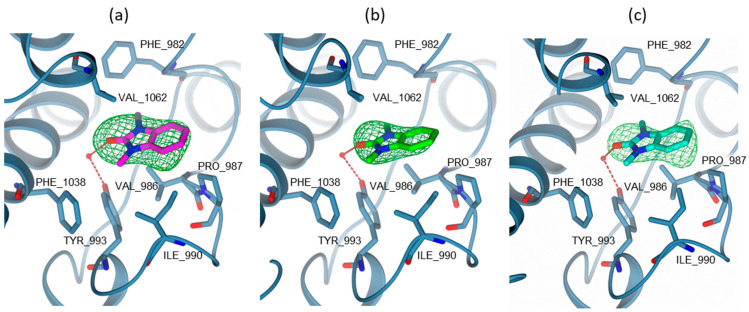
KAc binding site view of TRIM33α PHD-BRD (steel blue cartoon and carbon atoms; residues in sticks) in complex with (**a**) **8** (PDB code: 8BD8—in sticks, magenta carbon atoms); (**b**) **9** (PDB code: 8BDY—in sticks, green carbon atoms); (**c**) **10** (PDB code: 8BD9—in sticks, cyan carbon atoms). In all figures, ligands are surrounded by the omit map (dark green mesh) contoured at 2.5 σ level. Water molecules are displayed as red spheres (arbitrary radius). Hydrogen bonds are represented as red dashed lines. Oxygen and nitrogen atoms are coloured red and blue, respectively.

The structure of TRIM33α PHD-BRD was solved in complex with three tested ligands, compounds **8**, **9**, and **10,** to resolutions ranging from 3.10 to 3.20 Å (Table S6 in Supporting Information). In all complexes, the crystal asymmetric unit contains one independent molecule, having an overall structure highly conserved with previously reported models [39]. The protein chain was completely modelled apart from the region, including residues 885–947 and 956–1049. In all models, the ligands occupy the KAc binding site. The binding mode of their shared 1,3-dimethyl-1,3-dihydro-2H-benzo[d]imidazol-2-one core is highly conserved, retaining the same interactions within the cavity (Figure 14). The carbonyl moiety in position 1 of **8**, **9**, and **10** is involved in a water-mediated interaction with Tyr993. The core of the molecules is stabilised by van der Waals interactions within the hydrophobic cavity lined by PHE982, VAL986, PRO987, ILE990, PHE1038, and VAL1062. In all complexes, the variable R-groups in position 7 are characterised by positional disorder (reasonably due to alkyl chain rotation and a high degree of exposure) and thus excluded from our models (Figure 14). The docking analysis also gives as an output different possible poses of these compounds in TRIM33α, and the difference is just the position of the chain. This is free to move and rotate and can interact via salt bridge both with the GLU981 (folded chain) and GLU984 (stretched chain). Detailed information on this regard is reported in the Supporting Information (Figure S7). 

The X-ray binding mode of the core of compounds **8**, **9**, and **10** confirms both the obtained docking analysis and relative water consideration (See Figure S8 in Supporting information for details). Moreover, also the binding of the TRIM24 compounds (**2** and **3**) to TRIM33α was tested via crystallography following the same protocol reported for compounds **8**, **9**, and **10**. However, in these cases, only the ligand-free TRIM33α protein was obtained. This is a further confirmation of both the HTRF results obtained and of the repurposing strategy presented.

## 3. Materials and Methods 

### 3.1. Computational Methods 

#### 3.1.1. Data Collection 

All the structural data used in this work are reported in the ELIOT platform: X-ray crystallographic structures of TRIM24 and TRIM33 used for this study come from the PDB and related information is reported in Supplementary Information (Table S2). During the development of ELIOT, starting from the structures, pockets were detected using the BioGPS approach [30]. The BioGPS approach is based on the software FLAP, which combines GRID molecular interactions fields (MIFs) and pharmacophoric fingerprints (the detailed procedure has been reported previously [24]).

#### 3.1.2. Pocket Cross-Relationship Analysis

In order to compare pockets in terms of their three-dimensional non-bonded interaction similarity and to identify cross-relationships, it was necessary first to characterise them using GRID molecular interaction fields, thus evaluating the type, strength, and direction of the interactions that a pocket is capable of making with a putative ligand. To achieve this, the GRID probes H, CRY, O, and N1 were used to compute the shape, hydrophobic/lipophilic interactions, H-bond acceptor interactions, and H-bond donor interactions for each pocket, respectively. These MIFs were then used to compare all the E3 ligase pockets using BioGPS. The computational comparison was made during ELIOT development, and more details about the procedure are already published [24,30]. As a result, the Tanimoto similarity of the four GRID MIFs was calculated (ranging from 0.0 to 1.0), and a “Global-Sum” score from the individual MIF similarities was calculated [30,58,59].

#### 3.1.3. Energetic Contribution of the Residue to the MIFs

The calculation of the energy contribution of the residue to the MIFs was performed using a GRID-based approach. For each residue and for each probe (CRY, N1, and O), the maximum interaction value found in the grid is calculated. Given a certain grid point and a given probe in that position, GRID reports the index of the atom of the protein that interacts in the maximum entity with the probe itself (there could be other contributions from other atoms, but these atoms are ignored). The considered atom is associated with the residue to which it belongs, and the various results are gradually accumulated for each grid point and each probe, associating each atom to the corresponding residue.

#### 3.1.4. Docking Analysis

The docking analysis was performed using GLIDE (Schrödinger release 2020-3)

- Protein files

To better compare the results, all the structures were aligned onto the TRIM24 structure (PDB code: 4yc9 [42]) (RMSD 33α vs 4yc9 0.752Å; RMSD 33β vs 4yc9 0.760 Å). The TRIM33α and TRIM33β receptor file was prepared from chain A of the crystal structure of the protein (PDB codes 3u5o [39] and 5mr8 [36], respectively). Water molecules were not reported in the crystal structure of TRIM33α, consequently, the conserved water was included from the aligned TRIM24 structures (PDB codes: 4yc9 [42], 4yat [42], 4zql [42], 3o35 [57], and 3o36 [57]). The receptor files were subjected to Protein Preparation Wizard (Schrödinger release 2020-3) with default settings, and the direct and water-mediated interactions were optimised. For grid generation, the co-crystallised ligand 4A8 from the aligned PDB code 4yat [42] was used as the centre of the grid. The grid size was chosen on the basis of the 4A8 ligand (dock ligands similar in size option). During the grid generation, a constraint for the interaction with the conserved water was added.

- Ligand files

The 2D sdf files of the ligand (**2**, **3**, **4**, **5**, **6**, **7**, **8**, **9**, and **10**) were processed using LigPrep with default settings (SchrödingerSchrödinger release 2020-3). Possible protomers at pH 7.4 were evaluated, and all the obtained protonation states were considered.

- Docking

GLIDE was used in standard precision (SP) mode. For TRIM33β, the constraint with the water interaction was not used while for TRIM33α docking both with and without the water constraint were performed. Simulations generated 20 poses per ligand, and the conformation with the best docking score was retained. However, for ligands **7**, **8**, **9**, and **10** all the obtained poses were visually evaluated and other possible poses were also considered due to the different conformation of the aliphatic chain.

#### 3.1.5. The WaterFLAP Analysis

The WaterFLAP uses GRID to predict water networks in binding sites (with or without ligands) and score the individual waters according to their enthalpic and entropic energies of binding. In detail, it uses the GRID OH2 probe along with the addition of the CRY and ENTR MIFs, automated with an iterative approach and several refinement steps. The ENTR MIF is generated by the OH2 probe to characterise the entropy of the water at each grid position. In each direction around the grid point and for a certain distance, the OH2 enthalpy of interaction is calculated. If the enthalpy of interaction is not becoming less favourable within a certain tolerance, a water molecule at that position could therefore move freely. If the enthalpy of interaction is becoming less favourable, the water is less free to move. In the first case, the water is more entropic; in the second case less entropic. The energy values for the predicted waters are therefore characterised in the context of the network of waters (OH2n), or as standard, without considering the other waters in the network (OH2s). Each predicted water is characterised by a score (DG_WAT) that combines the OH2n, OH2s, CRY, and ENTR scores into a relative free energy binding score. Therefore, positive energies therefore indicate waters that would prefer to be displaced into solvent, whereas negative scores indicate happier waters. Scores of <−2 kcal/mol indicate waters where care should be taken if trying to displace them. A simple classification scheme has been devised to simplify analysis and highlight the important waters:

- Blue—happy (DG_WAT < −2.0 kcal);
- Grey—bulk-like (−2.0 kcal ≤ DG_WAT < 1.5 kcal);
- Yellow—unhappy (1.5 kcal ≤ DG_WAT < 3.0 kcal);
- Red—very unhappy (DG_WAT ≥ 3.0 kcal).

Here, ligands were included within the protein as part of the input receptor for the analysis in order to predict the water placement and energy considering the ligand influence and identifying possible structural waters which interact with it.

### 3.2. Synthetic Chemistry

Compounds **4**, **5**, **6**, **7**, **8**, **9**, and **10** were synthesised in house. Their synthetic procedures and characterisation are reported in the Supplementary Information.

### 3.3. HTRF Assay

HTRF binding experiments were performed in 20 µL of total assay volume in 384 well plates in accordance with the assay protocol reported by the manufacturer. Plates were incubated at room temperature for 1 h, 3 h, and overnight (ON) and a fluorescence emission signal at 620 and 665 nm was detected. Negative controls (CTRL-) for each assay were obtained by not adding the GST protein in order to have a non-specific signal to calculate either the HTRF ∆ratio (= specific signal) and the assay window (= S/B = HTRF ratio positive/HTRF ratio negative) for each peptide concentration. The S/B ratio is proportional to the protein/peptide interactions. Additional information about reagents, peptides, procedure, and data analysis are reported in the Supplementary Information.

### 3.4. Crystallographic Studies

Methodology for the protein expression, purification, crystallisation, and data collection are reported in detail in the Supplementary Information.

## 4. Conclusions

In conclusion, we have reported an example of possible applications of our ELIOT [24] platform for the identification of ligands of a novel E3 ligase for PROTAC design. The presented workflow starts from cross-relationship data available in ELIOT and demonstrates one of its possible applications. In particular, the potential of the GRID MIFs approach for the characterisation and comparison of pockets in order to expand the pool of the E3 ligases used for PROTAC is reported. The reported case study of the cross-relationship between the KAc binding site of the E3 ligases TRIM24 and TRIM33 was identified. Exploiting this cross-relationship, we showed how it is possible to design a scaffold-repurposing strategy. In particular, we used GRID MIFs to perform a deeper computational analysis of the TRIM24 and TRIM33 binding sites offering a different point of view than the more common one based on the sequence similarity. The scaffold-repurposing strategy was designed to analyse the interactions and the binding mode of the known X-ray crystallographic ligands of TRIM24. Comparing the energetic contribution to the MIFs of the key interacting residues in TRIM24 with the corresponding residues in TRIM33 pocket, similarities and differences were highlighted. The former was used to identify a common scaffold (compound **5**) between the TRIM24 ligands, while the latter was to identify ligands already reported in the literature with that scaffold (or similar, compound **6**) and specific substituents for TRIM33. Once identified in the literature, compounds with these characteristics (compounds **7**, **8**, **9**, and **10**) had a docking analysis performed. Both the TRIM33 isoforms were considered with a particular interest in the TRIM33α isoform. This is more challenging due to its non-canonical BRD, its wider KAc pocket, and its asparagine residue oriented out of the site. However, considering the presence of a conserved water molecule in the bottom of the KAc pocket of most BRDs and by including it within the TRIM33 structures to bias the docking, consistent poses with the known X-ray binding mode of TRIM24 ligands were obtained both for TRIM33α and TRIM33β. It is important to note that while similar compounds were already docked into the binding site of TRIM33β (it conserves the canonical KAc pocket), similar docking poses have never been reported in TRIM33α before. Similarly, using WaterFLAP for the first time, an analysis of the water molecules was performed by predicting both their placement in the TRIM33-ligand complex and their relative free energy from a combination of their enthalpic and entropic contributions. In this way, we have highlighted the key role of the conserved water molecule in ligand binding to TRIM33α and the presence of another possible water molecule, equally important, which in TRIM33α exactly occupies the empty spot left from the movement of the asparagine residue, resulting in another possible anchoring point for the ligand. To validate the computational studies and the used repurposing strategy, the identified TRIM33 ligands were synthesised and their binding verified via HTRF assay. The HTRF results suggest that both scaffold **5** and **6** and compounds **7**, **8**, **9**, and **10** bind TRIM33α and TRIM33β, while the known TRIM24 ligands **2**, **3**, and **4** did not bind. Although it was not possible to determine the IC_50_ via HTRF assay, keeping in mind the aim of the work, the decrease of the HTRF signal was nevertheless satisfactory and encouraging enough for us to conduct crystallographic studies. Therefore, the structure of TRIM33α PHD-BRD was solved in a complex with three of the tested ligands, compounds **8**, **9**, and **10,** with resolutions ranging from 3.10 to 3.20 Å. The crystals obtained are the first ever reported for a complex of TRIM33α with its ligands (even if only the core is visible). They strengthen the weak experimental HTRF data obtained, confirm the docking proposed for the TRIM33 ligands and the study carried out on the water molecules and validate the scaffold-repurposing procedure based on the GRID MIFs approach used in ELIOT.

This extensive work demonstrates how, starting from ELIOT and exploiting the MIFs approach, it is possible to design an optimised scaffold-repurposing strategy aimed at the discovery and optimisation of ligands for new E3 ligases. Thus, this approach may expand the enormous therapeutic potential offered by the targeted protein degradation field by exploiting PROTAC technology. Moreover, considering the therapeutic interest that surrounds TRIM33, the reported case study uses a different approach than the one used by Sekirnik et al. [36] but confirms part of their data and opens the way forward to the optimization of the identified TRIM33 compounds, in order to design PROTACs exploiting the TRIM33 E3 ligase activity.

## Data Availability

All reported structural 3D information (protein structures, pockets, MIFs, ligands, tissue, pathologies, and cross-relationship) can be found at https://eliot.moldiscovery.com previous registration (trial licenses are available). The BioGPS software, containing executables for pocket detection, pocket characterisation, and pocket comparison is available from https://www.moldiscovery.com/, and trial licenses are available to both commercial and academic users. Crystal structure final coordinates and structure factors are available in the PDB (www.rcsb.org—accessed on 21 October 2022) under the codes 8BD8 (TRIM33α PHD-BRD—8), 8BDY (TRIM33α PHD-BRD—9), and 8BD9 (TRIM33α PHD-BRD—10).

## Supplementary Materials

The supporting information can be downloaded at: https://www.mdpi.com/article/10.3390/ijms232214218/s1. References [60,61,62,63,64,65,66,67,68,69,70,71,72,73,74] are cited in the supplementary materials.
