# Supplementary material for: Exploiting ELIOT for Scaffold-Repurposing Opportunities: TRIM33 a Possible Novel E3 Ligase to Expand the Toolbox for PROTAC Design
Source: Int J Mol Sci. 2022 Nov 17;23(22):14218. doi: 10.3390/ijms232214218 (PMC9698485; doi:10.3390/ijms232214218)
Supplement: Supplementary file 1 [file ijms-23-14218-s001.zip › ijms-2024121-supplementary.pdf]

Exploiting ELIOT for scaffold-repurposing opportunities: TRIM33 a possible novel E3 ligase to expand the toolbox for PROTAC design

Tommaso Palomba[1], Giusy Tassone[2], Carmine Vacca[1], Matteo Bartalucci[1], Aurora Valeri[3], Cecilia Pozzi[2], Simon Cross[4], Lydia Siragusa[3,4]* and Jenny Desantis[1]*

1 Department of Chemistry, Biology, and Biotechnology, University of Perugia, Via Elce di Sotto, 8, 06132 Perugia, Italy
2 Department of Biotechnology, Chemistry and Pharmacy, University of Siena, Via Aldo Moro 2, I-53100 Siena, Italy
3 Molecular Horizon srl, Via Montelino, 20, 06084, Bettona (PG), Italy
4 Molecular Discovery Ltd., Kinetic Business Centre, Theobald Street, Elstree, Borehamwood, Hertfordshire WD6 4PJ, U.K.
* Correspondence: jenny.desantis@unipg.it; lydia@moldiscovery.com;
Tel.: +39 075 5855262 (J.D.); +39 075 5855550 (L.S.)

Supporting Information

Contents

Integrative results 3
1.1 TRIM33 BioGRID interactors 3
1.2 Information of the structures present in the PDB and used in this work 12
1.3 Comparison of the main contact regions of the KAc pocket 13
1.4 Energetic scanning of residues 13
1.5 Consideration about the X-ray conserved water molecule 16
1.6 Docking in TRIM33α without the conserved water molecule 17
1.7 Docking in TRIM33β 18
1.8 Comparison between the canonical BRD of TRIM24 and the not-canonical BRD of TRIM28 19
1.9 K_d determination of the Peptide/TRIM systems 20
1.10 Possible chain conformations from docking analysis 21
1.11 TRIM33α ligands: Experimental binding mode vs Predicted pose 22

Experimental section **23**
2.1 HTRF assay 23
HTRF reagents 23
Proteins, peptides and ligands 23

Filter set 24
Kit and peptide selection procedure 24
Peptide titration procedure 25
Competition binding procedure 25
Data analysis 25
2.2 Chemistry 27
General Synthetic Chemistry Methods 27
1,3-Dimethyl-5-nitro-1H-benzo[d]imidazol-2(3H)-one (S11) 27
5-Amino-1,3-dimethyl-1H-benzo[d]imidazol-2(3H)-one (5) 28
5-Amino-6-bromo-1,3-dimethyl-1H-benzo[d] midazole-2(3H)-one (S12) 28
N-(6-Bromo-1,3-dimethyl-2-oxo-2,3-dihydro-1H-benzo[d]imidazol-5-yl)-2,2,2-trifluoroacetamide (S13) 28
5-Amino-1,3-dimethyl-6-(3-propoxyphenoxy)-1H-benzo[d]imidazol-2(3H)-one (S15) 28
N-(1,3-Dimethyl-2-oxo-6-(3-propoxyphenoxy)-2,3-dihydro-1H-benzo[d]imidazol-5-yl)-3,4-dimethoxybenzenesulfonamide (4) 29
3-propoxyphenol (S14) 29
2-Oxo-2,3-dihydro-1H-benzo[d]imidazole-5-carbonitrile (S16) 29
1,3-Dimethyl-2-oxo-2,3-dihydro-1H-benzo[d]imidazole-5-carbonitrile 30
5-(Aminomethyl)-1,3-dimethyl-1H-benzo[d]imidazol-2(3H)-one (6) 30
General Procedure A for reductive amination 30
5-(((3-(Dimethylamino)propyl)amino)methyl)-1,3-dimethyl-1H-benzo[d]imidazol-2(3H)-one (7) . 30
5-(((3-(Diethylamino)propyl)amino)methyl)-1,3-dimethyl-1H-benzo[d]imidazol-2(3H)-one (8) 31
5-(((4-(Dimethylamino)butyl)amino)methyl)-1,3-dimethyl-1H-benzo[d]imidazol-2(3H)-one (9) 31
1,3-Dimethyl-5-(((3-(pyrrolidin-1-yl)propyl)amino)methyl)-1H-benzo[d]imidazol-2(3H)-one (10). 31
2.3 Crystallographic studies 31
TRIM33α PHD-BRD expression and purification 31
Crystallization 32
Data collection, structure solution and refinement 32

Bibliography **34**

Integrative results

1.1 TRIM33 BioGRID interactors

Table S1. TRIM33 interactors exported from BioGRID. Interactors from H. sapiens, M. musculus, MERS-CoV, R. norvegiocus and X. laevis are reported.

Interactor	Organism	Aliases	Description
ACY1	H. sapiens	ACY1D, ACY-1, HEL-S-5	aminoacylase 1
ADCK3	H. sapiens	COQ8, SCAR9, ARCA2, PP265, CABC1, COQ10D4	aarF domain containing kinase 3
ANAPC1	H. sapiens	MCPR, APC1, TSG24	anaphase promoting complex subunit 1
ANAPC4	H. sapiens	APC4	anaphase promoting complex subunit 4
ANAPC5	H. sapiens	APC5	anaphase promoting complex subunit 5
ANAPC7	H. sapiens	APC7	anaphase promoting complex subunit 7
ANGEL1	H. sapiens	Ccr4e, KIAA0759	angel homolog 1 (Drosophila)
ANKRD28	H. sapiens	PITK, PPP1R65	ankyrin repeat domain 28
AOX1	H. sapiens	AO, AOH1	aldehyde oxidase 1
AR	H. sapiens	KD, TFM, AIS, SBMA, DHTR, NR3C4, HYSP1, SMAX1, HUMARA, RP11-383C12.1	androgen receptor
ARF5	H. sapiens	-	ADP-ribosylation factor 5
ARID1B	H. sapiens	OSA2, P250R, DAN15, MRD12, 6A3-5, BRIGHT, BAF250B, ELD/OSA1, RP11-419L10.1	AT rich interactive domain 1B (SWI1-like)
ASF1A	H. sapiens	CIA, CGI-98, HSPC146, RP3-329L24.1	anti-silencing function 1A histone chaperone
ASS1	H. sapiens	ASS, CTLN1, RP11-618A20.2	argininosuccinate synthase 1
B4GALT2	H. sapiens	B4Gal-T2, B4Gal-T3, beta4Gal-T2	UDP-Gal:betaGlcNAc beta 1,4-galactosyltransferase, polypeptide 2
BAG2	H. sapiens	BAG-2, dJ417I1.2, RP3-496N17.2	BCL2-associated athanogene 2
BCKDHA	H. sapiens	MSU, MSUD1, OVD1A, BCKDE1A	branched chain keto acid dehydrogenase E1, alpha polypeptide
BCKDHB	H. sapiens	E1B, dJ279A18.1, RP1-279A18.1	branched chain keto acid dehydrogenase E1, beta polypeptide
BRCA1	H. sapiens	IRIS, PSCP, FANCS, RNF53, BRCC1, PNCA4, BRCAI, PPP1R53, BROVCA1	breast cancer 1, early onset
BRD1	H. sapiens	BRL, BRPF2, BRPF1, RP3-522J7.4	bromodomain containing 1
BRD4	H. sapiens	CAP, MCAP, HUNKI, HUNK1	bromodomain containing 4
BTBD18	H. sapiens	hCG_1730474	BTB (POZ) domain containing 18
BUB1B	H. sapiens	MVA1, SSK1, Bub1A, MAD3L, BUBR1, hBUBR1, BUB1beta	BUB1 mitotic checkpoint serine/threonine kinase B
C12ORF74	H. sapiens	-	chromosome 12 open reading frame 74
C17ORF53	H. sapiens	-	chromosome 17 open reading frame 53

C22ORF15	H. sapiens	N27C7-3	chromosome 22 open reading frame 15
C9ORF78	H. sapiens	HCA59, HSPC220, bA409K20.3	chromosome 9 open reading frame 78
CALM1	H. sapiens	caM, CAMI, PHKD, DD132, CPVT4, CALML2	calmodulin 1 (phosphorylase kinase, delta)
CALU	H. sapiens	-	calumenin
CAV1	H. sapiens	CGL3, PPH3, LCCNS, VIP21, BSCL3, MSTP085	caveolin 1, caveolae protein, 22kDa
CBFA2T3	H. sapiens	ETO2, MTG16, MTGR2, ZMYND4	core-binding factor, runt domain, alpha subunit 2; translocated to, 3
CBX3	H. sapiens	HECH, HP1-GAMMA, HP1Hs-gamma	chromobox homolog 3
CCNA2	H. sapiens	CCN1, CCNA	cyclin A2
CCT8L2	H. sapiens	CESK1	chaperonin containing TCP1, subunit 8 (theta)-like 2
CDC16	H. sapiens	APC6, CUT9, ANAPC6, RP11-569D9.4	cell division cycle 16
CDC20	H. sapiens	CDC20A, p55CDC, bA276H19.3	cell division cycle 20
CDC23	H. sapiens	APC8, CUT23, ANAPC8	cell division cycle 23
CDC27	H. sapiens	HNUC, NUC2, APC3, H-NUC, ANAPC3, CDC27Hs, D0S1430E, D17S978E	cell division cycle 27
CDK9	H. sapiens	TAK, CTK1, C-2k, CDC2L4, PITALRE, RP11-228B15.5	cyclin-dependent kinase 9
CEP78	H. sapiens	IP63, C9orf81, RP11-336N8.5	centrosomal protein 78kDa
CHAMP1	H. sapiens	CAMP, CHAMP, ZNF828, C13orf8	chromosome alignment maintaining phosphoprotein 1
CHD1L	H. sapiens	CHDL, ALC1	chromodomain helicase DNA binding protein 1-like
CHEK1	H. sapiens	CHK1	checkpoint kinase 1
CLTC	H. sapiens	Hc, CHC, CHC17, CLH-17, CLTCL2	clathrin, heavy chain (Hc)
CNKSR1	H. sapiens	CNK, CNK1	connector enhancer of kinase suppressor of Ras 1
CREBBP	H. sapiens	CBP, RSTS, KAT3A	CREB binding protein
CRYAA	H. sapiens	HSPB4, CRYA1, CTRCT9	crystallin, alpha A
CRYAB	H. sapiens	MFM2, HSPB5, CTPP2, CRYA2, CMD1II, CTRCT16, HEL-S-101	crystallin, alpha B
CTNNA1	H. sapiens	CAP102	catenin (cadherin-associated protein), alpha 1, 102kDa
CTNNB1	H. sapiens	CTNNB, MRD19, armadillo, OK/SW-cl.35	catenin (cadherin-associated protein), beta 1, 88kDa
CUL3	H. sapiens	CUL-3, PHA2E	cullin 3
CYLD	H. sapiens	SBS, TEM, EAC, MFT, CDMT, MFT1, BRSS, CYLD1, USPL2, CYLDI, ...	cylindromatosis (turban tumor syndrome)
DBT	H. sapiens	E2, E2B, BCATE2, BCKADE2, BCKAD-E2, RP11-305E17.3	dihydrolipoamide branched chain transacylase E2
DCAF15	H. sapiens	C19orf72	DDB1 and CUL4 associated factor 15
DCAF7	H. sapiens	AN11, WDR68, HAN11, SWAN-1	DDB1 and CUL4 associated factor 7
DGKE	H. sapiens	DGK, DAGK5, DAGK6, NPHS7	diacylglycerol kinase, epsilon 64kDa
DHX33	H. sapiens	DDX33	DEAH (Asp-Glu-Ala-His) box polypeptide 33

DNAJC10	H. sapiens	MTHr, JPDI, ERdj5, PDIA19, UNQ495/PRO1012	DnaJ (Hsp40) homolog, subfamily C, member 10
DPP7	H. sapiens	QPP, DPP2, DPPII	dipeptidyl-peptidase 7
EDEM3	H. sapiens	C1orf22	ER degradation enhancer, mannosidase alpha-like 3
EGFR	H. sapiens	ERBB, HER1, mENA, PIG61, ERBB1, NISBD2	epidermal growth factor receptor
EIF3A	H. sapiens	p185, EIF3, P167, p180, TIF32, EIF3S10, eIF3-p170, eIF3-theta	eukaryotic translation initiation factor 3, subunit A
ELAVL1	H. sapiens	HUR, Hua, MelG, ELAV1	ELAV like RNA binding protein 1
ELF4	H. sapiens	MEF, ELFR, RP3-510O21.2	E74-like factor 4 (ets domain transcription factor)
ELF5	H. sapiens	ESE2	E74-like factor 5 (ets domain transcription factor)
EPAS1	H. sapiens	HLF, MOP2, HIF2A, ECYT4, PASD2, bHLHe73	endothelial PAS domain protein 1
ERC1	H. sapiens	ELKS, Cast2, ERC-1, RAB6IP2	ELKS/RAB6-interacting/CAST family member 1
ERG	H. sapiens	p55, erg-3	v-ets avian erythroblastosis virus E26 oncogene homolog
ESR1	H. sapiens	ER, ESR, Era, ESRA, ESTRR, NR3A1, RP1-130E4.1	estrogen receptor 1
ESR2	H. sapiens	Erb, ESRB, NR3A2, ESTRB, ER-BETA, ESR-BETA	estrogen receptor 2 (ER beta)
EWSR1	H. sapiens	EWS, bK984G1.4, AC002059.7	EWS RNA-binding protein 1
EZH2	H. sapiens	WVS, ENX1, WVS2, KMT6, EZH1, ENX-1, EZH2b, KMT6A	enhancer of zeste 2 polycomb repressive complex 2 subunit
FAM53C	H. sapiens	C5orf6	family with sequence similarity 53, member C
FBN2	H. sapiens	CCA, DA9, EOMD	fibrillin 2
FGA	H. sapiens	Fib2	fibrinogen alpha chain
FHL3	H. sapiens	SLIM2	four and a half LIM domains 3
FHL5	H. sapiens	ACT, dJ393D12.2, RP3-393D12.2	four and a half LIM domains 5
FOS	H. sapiens	p55, AP-1, C-FOS	FBJ murine osteosarcoma viral oncogene homolog
FOXA1	H. sapiens	HNF3A, TCF3A	forkhead box A1
FOXF2	H. sapiens	FKHL6, FREAC2, FREAC-2	forkhead box F2
FOXI1	H. sapiens	HFH3, HFH-3, FKH10, FREAC6, FKHL10, FREAC-6	forkhead box I1
FZR1	H. sapiens	FZR, FZR2, HCDH, CDH1, HCDH1, CDC20C	fizzy/cell division cycle 20 related 1 (Drosophila)
GCDH	H. sapiens	GCD, ACAD5	glutaryl-CoA dehydrogenase
GCHFR	H. sapiens	P35, GFRP, HsT16933	GTP cyclohydrolase I feedback regulator
GFOD1	H. sapiens	ADG-90, C6orf114, RP11-501I19.1	glucose-fructose oxidoreductase domain containing 1
GGCX	H. sapiens	VKCFD1	gamma-glutamyl carboxylase
GNB2L1	H. sapiens	HLC7, HLC-7, H12.3, PIG21, RACK1, Gnb2-rs1	guanine nucleotide binding protein (G protein), beta polypeptide 2-like 1

GPX1	H. sapiens	GPXD, GSHPX1	glutathione peroxidase 1
GSPT1	H. sapiens	GST1, eRF3a, ETF3A, 551G9.2	G1 to S phase transition 1
HAO2	H. sapiens	HAOX2, GIG16, RP5-871G17.1	hydroxyacid oxidase 2 (long chain)
HDAC6	H. sapiens	HD6, JM21, CPBHM, PPP1R90	histone deacetylase 6
HIST1H3A	H. sapiens	H3FA, H3/A	histone cluster 1, H3a
HNF4A	H. sapiens	TCF, MODY, HNF4, FRTS4, TCF14, MODY1, NR2A1, HNF4a9, HNF4a8, HNF4a7, …	hepatocyte nuclear factor 4, alpha
HOXC5	H. sapiens	HOX3, CP11, HOX3D	homeobox C5
HSPA2	H. sapiens	HSP70-2, HSP70-3	heat shock 70kDa protein 2
HSPA8	H. sapiens	LAP1, NIP71, LAP-1, HSC70, HSC71, HSC54, HSP71, HSP73, HEL-33, HSPA10, …	heat shock 70kDa protein 8
HSPB2	H. sapiens	MKBP, HSP27, Hs.78846, LOH11CR1K	heat shock 27kDa protein 2
HSPH1	H. sapiens	HSP105, HSP105B, HSP105A, NY-CO-25, RP11-173P16.1	heat shock 105kDa/110kDa protein 1
IMMP1L	H. sapiens	IMP1, IMP1-LIKE	IMP1 inner mitochondrial membrane peptidase-like (S. cerevisiae)
JUN	H. sapiens	AP1, AP-1, c-Jun	jun proto-oncogene
KCTD17	H. sapiens	RP5-1170K4.4	potassium channel tetramerization domain containing 17
KDM5C	H. sapiens	SMCX, MRXJ, XE169, MRXSJ, MRX13, MRXSCJ, JARID1C, DXS1272E, RP11-258C19.2	lysine (K)-specific demethylase 5C
KIAA1429	H. sapiens	MSTP054, fSAP121	KIAA1429
KIAA1551	H. sapiens	C12orf35	KIAA1551
KIF11	H. sapiens	EG5, HKSP, MCLMR, TRIP5, KNSL1	kinesin family member 11
KIF5B	H. sapiens	KNS, UKHC, KINH, KNS1, HEL-S-61	kinesin family member 5B
KLC1	H. sapiens	KLC, KNS2, KNS2A, RP11-73M18.7	kinesin light chain 1
KLC2	H. sapiens	-	kinesin light chain 2
KLF8	H. sapiens	BKLF3, ZNF741, RP13-1021K9.1	Kruppel-like factor 8
KLHL20	H. sapiens	KLEIP, KHLHX, KLHLX, RP3-383J4.3	kelch-like family member 20
KLK9	H. sapiens	KLKL3, KLK-L3	kallikrein-related peptidase 9
KPNA1	H. sapiens	RCH2, SRP1, IPOA5, NPI-1	karyopherin alpha 1 (importin alpha 5)
KPNA6	H. sapiens	KPNA7, IPOA7, RP4-622L5.1	karyopherin alpha 6 (importin alpha 7)
KRT37	H. sapiens	K37, HA7, KRTHA7	keratin 37
KRT38	H. sapiens	HA8, hHa8, KRTHA8	keratin 38
KRT39	H. sapiens	K39, KA35, CK-39	keratin 39
LARP1	H. sapiens	LARP	La ribonucleoprotein domain family, member 1
LCE3D	H. sapiens	LEP16, SPRL6B, SPRL6A	late cornified envelope 3D
LDB1	H. sapiens	NLI, CLIM2	LIM domain binding 1
LHX4	H. sapiens	CPHD4	LIM homeobox 4
LHX6	H. sapiens	LHX6.1, RP11-498E2.6	LIM homeobox 6
LMNB1	H. sapiens	LMN, LMN2, ADLD, LMNB	lamin B1

LMO2	H. sapiens	TTG2, RHOM2, RBTN2, RBTNL1	LIM domain only 2 (rhombotin-like 1)
LOXL4	H. sapiens	LOXC	lysyl oxidase-like 4
LRP2	H. sapiens	DBS, GP330	low density lipoprotein receptor-related protein 2
LRPPRC	H. sapiens	LSFC, GP130, LRP130, CLONE-23970	leucine-rich pentatricopeptide repeat containing
LURAP1	H. sapiens	LRP35A, LRAP35a, C1orf190	leucine rich adaptor protein 1
LY6G5B	H. sapiens	G5b, C6orf19, DADB-127H9.9	lymphocyte antigen 6 complex, locus G5B
LYL1	H. sapiens	bHLHa18	lymphoblastic leukemia associated hematopoiesis regulator 1
MAD2L1	H. sapiens	MAD2, HSMAD2	MAD2 mitotic arrest deficient-like 1 (yeast)
MAGEA9	H. sapiens	CT1.9, MAGE9	melanoma antigen family A, 9
MAPK6	H. sapiens	ERK3, PRKM6, p97MAPK, HsT17250	mitogen-activated protein kinase 6
MCM3	H. sapiens	P1.h, RLFB, HCC5, P1-MCM3, RP1-108C2.3	minichromosome maintenance complex component 3
MDC1	H. sapiens	NFBD1, DAAP-285E11.7	mediator of DNA-damage checkpoint 1
METTL21B	H. sapiens	FAM119B	methyltransferase like 21B
MLKL	H. sapiens	hMLKL	mixed lineage kinase domain-like
MTA1	H. sapiens	-	metastasis associated 1
MYC	H. sapiens	MYCC, MRTL, c-Myc, bHLHe39	v-myc avian myelocytomatosis viral oncogene homolog
MYOD1	H. sapiens	PUM, MYF3, MYOD, bHLHc1	myogenic differentiation 1
NAA11	H. sapiens	ARD2, hARD2, ARD1B	N(alpha)-acetyltransferase 11, NatA catalytic subunit
NANOG	H. sapiens	-	Nanog homeobox
NCOA3	H. sapiens	AIB1, RAC3, ACTR, SRC3, pCIP, CTG26, SRC-3, AIB-1, TNRC16, TNRC14, ...	nuclear receptor coactivator 3
NCOR1	H. sapiens	N-CoR, TRAC1, N-CoR1, hN-CoR, PPP1R109	nuclear receptor corepressor 1
NELL2	H. sapiens	NRP2	NEL-like 2 (chicken)
NID2	H. sapiens	NID-2	nidogen 2 (osteonidogen)
NIN	H. sapiens	SCKL7	ninein (GSK3B interacting protein)
NLGN3	H. sapiens	HNL3	neuroligin 3
NSMCE4A	H. sapiens	NS4EA, NSE4A, PP4762, C10orf86	non-SMC element 4 homolog A (S. cerevisiae)
NT5DC2	H. sapiens	-	5'-nucleotidase domain containing 2
NTRK1	H. sapiens	TRK, MTC, TRKA, TRK1, Trk-A, p140-TrkA	neurotrophic tyrosine kinase, receptor, type 1
NUP35	H. sapiens	MP44, NP44, NUP53, MP-44	nucleoporin 35kDa
NUP50	H. sapiens	NPAP60, NPAP60L, CTA-268H5.7	nucleoporin 50kDa
NUP62	H. sapiens	p62, IBSN, SNDI	nucleoporin 62kDa
OSBP	H. sapiens	OSBP1	oxysterol binding protein
PBK	H. sapiens	SPK, TOPK, CT84, HEL164, Nori-3	PDZ binding kinase
PDIA4	H. sapiens	ERP72, ERP70, ERp-72	protein disulfide isomerase family A, member 4

PELO	H. sapiens	CGI-17, PRO1770	pelota homolog (Drosophila)
PER1	H. sapiens	PER, hPER, RIGUI	period circadian clock 1
PEX5	H. sapiens	PXR1, PBD2A, PTS1R, PBD2B, PTS1-BP	peroxisomal biogenesis factor 5
PIP4K2C	H. sapiens	PIP5K2C	phosphatidylinositol-5-phosphate 4-kinase, type II, gamma
PJA2	H. sapiens	RNF131, Neurodap1	praja ring finger 2, E3 ubiquitin protein ligase
PLEKHA4	H. sapiens	PEPP1	pleckstrin homology domain containing, family A (phosphoinositide binding specific) member 4
PML	H. sapiens	MYL, RNF71, TRIM19, PP8675	promyelocytic leukemia
PNMA1	H. sapiens	MA1	paraneoplastic Ma antigen 1
PNMA2	H. sapiens	MA2, MM2, RGAG2	paraneoplastic Ma antigen 2
POGZ	H. sapiens	ZNF635, ZNF280E, ZNF635m, RP11-806J18.2	pogo transposable element with ZNF domain
POLR2C	H. sapiens	RPB3, RPB31, hRPB33, hsRPB3, A-152E5.7	polymerase (RNA) II (DNA directed) polypeptide C, 33kDa
PRR20A	H. sapiens	PRR20	proline rich 20A
PSMB7	H. sapiens	Z, RP11-101K10.7	proteasome (prosome, macropain) subunit, beta type, 7
PSME3	H. sapiens	Ki, PA28G, REG-GAMMA, PA28gamma, HEL-S-283, PA28-gamma	proteasome (prosome, macropain) activator subunit 3 (PA28 gamma; Ki)
PTPN11	H. sapiens	CFC, NS1, SHP2, BPTP3, PTP2C, PTP-1D, SH-PTP3, SH-PTP2	protein tyrosine phosphatase, non-receptor type 11
PYCRL	H. sapiens	-	pyrroline-5-carboxylate reductase-like
QPRT	H. sapiens	QPRTase, HEL-S-90n	quinolinate phosphoribosyltransferase
RABGGTB	H. sapiens	GGTB, RP4-682C21.3	Rab geranylgeranyltransferase, beta subunit
RAD50	H. sapiens	NBSLD, RAD502, hRad50	RAD50 homolog (S. cerevisiae)
RBMX	H. sapiens	RNMX, HNRPG, RBMXP1, RBMXRT, HNRNPG, hnRNP-G, RP11-1114A5.1	RNA binding motif protein, X-linked
RCN1	H. sapiens	RCN, RCAL, PIG20, HEL-S-84	reticulocalbin 1, EF-hand calcium binding domain
RCN2	H. sapiens	E6BP, ERC55, ERC-55, TCBP49	reticulocalbin 2, EF-hand calcium binding domain
RFC4	H. sapiens	A1, RFC37	replication factor C (activator 1) 4, 37kDa
RNF31	H. sapiens	HOIP, ZIBRA	ring finger protein 31
RPAP3	H. sapiens	-	RNA polymerase II associated protein 3
RPS6KA6	H. sapiens	RSK4, PP90RSK4	ribosomal protein S6 kinase, 90kDa, polypeptide 6
RRAGC	H. sapiens	RAGC, GTR2, TIB929	Ras-related GTP binding C
RRM1	H. sapiens	R1, RR1, RIR1	ribonucleotide reductase M1
RUNX1	H. sapiens	AML1, CBFA2, EVI-1, AMLCR1, PEBP2aB, AML1-EVI-1	runt-related transcription factor 1

RUNX1T1	H. sapiens	CDR, ETO, MTG8, AML1T1, ZMYND2, CBFA2T1	runt-related transcription factor 1; translocated to, 1 (cyclin D-related)
SAT1	H. sapiens	SAT, DC21, SSAT, KFSD, KFSDX, SSAT-1	spermidine/spermine N1-acetyltransferase 1
SCO2	H. sapiens	MYP6, SCO1L, CEMCOX1	SCO2 cytochrome c oxidase assembly protein
SDF4	H. sapiens	Cab45, SDF-4, RP5-902P8.6	stromal cell derived factor 4
SHARPIN	H. sapiens	SIPL1, PSEC0216	SHANK-associated RH domain interactor
SHFM1	H. sapiens	ECD, SEM1, DSS1, SHFD1, SHSF1, Shfdg1	split hand/foot malformation (ectrodactyly) type 1
SKI	H. sapiens	SGS, SKV	SKI proto-oncogene
SKIL	H. sapiens	SNO, SnoA, SnoN, SnoI	SKI-like proto-oncogene
SKIV2L	H. sapiens	HLP, SKI2, 170A, DDX13, SKIV2, THES2, SKI2W, DADB-112B14.3	superkiller viralicidic activity 2-like (S. cerevisiae)
SLFN11	H. sapiens	SLFN8/9	schlafen family member 11
SLK	H. sapiens	LOSK, STK2, se20-9, bA16H23.1	STE20-like kinase
SMAD2	H. sapiens	JV18, MADR2, MADH2, hMAD-2, hSMAD2, JV18-1	SMAD family member 2
SMAD3	H. sapiens	LDS3, MADH3, LDS1C, JV15-2, HSPC193, HsT17436	SMAD family member 3
SMAD4	H. sapiens	JIP, DPC4, MYHRS, MADH4	SMAD family member 4
SMARCA4	H. sapiens	BRG1, SWI2, SNF2, RTPS2, MRD16, SNF2L4, SNF2LB, BAF190, hSNF2b, BAF190A	SWI/SNF related, matrix associated, actin dependent regulator of chromatin, subfamily a, member 4
SMARCC1	H. sapiens	Rsc8, SRG3, SWI3, CRACC1, BAF155	SWI/SNF related, matrix associated, actin dependent regulator of chromatin, subfamily c, member 1
SMARCC2	H. sapiens	Rsc8, CRACC2, BAF170	SWI/SNF related, matrix associated, actin dependent regulator of chromatin, subfamily c, member 2
SMS	H. sapiens	SRS, SpS, MRSR, SPMSY	spermine synthase
SOD1	H. sapiens	SOD, ALS, IPOA, ALS1, hSod1, HEL-S-44, homodimer	superoxide dismutase 1, soluble
SOHLH1	H. sapiens	TEB2, NOHLH, SPATA27, bHLHe80, C9orf157, bA100C15.3, RP11-100C15.3	spermatogenesis and oogenesis specific basic helix-loop-helix 1
SOX17	H. sapiens	VUR3	SRY (sex determining region Y)-box 17
SOX2	H. sapiens	ANOP3, MCOPS3	SRY (sex determining region Y)-box 2
SP100	H. sapiens	lysp100b	SP100 nuclear antigen
SP7	H. sapiens	OSX, OI12, OI11, osterix	Sp7 transcription factor
SSB	H. sapiens	La, LARP3, La/SSB	Sjogren syndrome antigen B (autoantigen La)
SSBP2	H. sapiens	SOSS-B2, HSPC116	single-stranded DNA binding protein 2
SSBP3	H. sapiens	SSDP, CSDP, SSDP1, RP5-997D24.2	single stranded DNA binding protein 3
SSBP4	H. sapiens	-	single stranded DNA binding protein 4

SSRP1	H. sapiens	T160, FACT, FACT80	structure specific recognition protein 1
SSSCA1	H. sapiens	p27	Sjogren syndrome/scleroderma autoantigen 1
ST13	H. sapiens	HIP, HOP, P48, SNC6, AAG2, HSPABP, HSPABP1, FAM10A1, PRO0786, FAM10A4, ...	suppression of tumorigenicity 13 (colon carcinoma) (Hsp70 interacting protein)
SUMO2	H. sapiens	Smt3A, SUMO3, SMT3B, HSMT3, SMT3H2	small ubiquitin-like modifier 2
SUPT16H	H. sapiens	CDC68, FACTP140, SPT16/CDC68	suppressor of Ty 16 homolog (S. cerevisiae)
TAL1	H. sapiens	SCL, TCL5, tal-1, bHLHa17	T-cell acute lymphocytic leukemia 1
TBL1X	H. sapiens	EBI, TBL1, SMAP55	transducin (beta)-like 1X-linked
TBL1XR1	H. sapiens	C21, IRA1, DC42, TBLR1	transducin (beta)-like 1 X-linked receptor 1
TCEB2	H. sapiens	ELOB, SIII	transcription elongation factor B (SIII), polypeptide 2 (18kDa, elongin B)
TCF12	H. sapiens	HEB, CRS3, HTF4, bHLHb20, HsT17266	transcription factor 12
TCF3	H. sapiens	E47, E2A, VDIR, ITF1, TCF-3, bHLHb21	transcription factor 3
TCF4	H. sapiens	SEF2, ITF2, E2-2, PTHS, TCF-4, SEF-2, ITF-2, SEF2-1, bHLHb19, SEF2-1B, ...	transcription factor 4
TERF2IP	H. sapiens	RAP1, DRIP5, PP8000	telomeric repeat binding factor 2, interacting protein
TEX13B	H. sapiens	TGC3B, TSGA5	testis expressed 13B
TGM7	H. sapiens	TGMZ	transglutaminase 7
THOC1	H. sapiens	P84, HPR1, P84N5	THO complex 1
THRSP	H. sapiens	S14, Lpgp, THRP, LPGP1, SPOT14	thyroid hormone responsive
TIGD5	H. sapiens	-	tigger transposable element derived 5
TIMM50	H. sapiens	TIM50, TIM50L, PRO1512	translocase of inner mitochondrial membrane 50 homolog (S. cerevisiae)
TMX3	H. sapiens	PDIA13, TXNDC10	thioredoxin-related transmembrane protein 3
TP53	H. sapiens	P53, LFS1, BCC7, TRP53	tumor protein p53
TP53BP1	H. sapiens	p202, 53BP1	tumor protein p53 binding protein 1
TPM3	H. sapiens	TRK, TM5, TM3, CFTD, TM-5, TM30, NEM1, CAPM1, hscp30, TPMsk3, ...	tropomyosin 3
TRAF2	H. sapiens	TRAP, TRAP3, MGC:45012	TNF receptor-associated factor 2
TRIM24	H. sapiens	TF1A, TIF1, PTC6, RNF82, TIF1A, hTIF1, TIF1ALPHA	tripartite motif containing 24
TRIM25	H. sapiens	EFP, Z147, ZNF147, RNF147	tripartite motif containing 25
TRIM28	H. sapiens	TF1B, KAP1, TIF1B, RNF96, PPP1R157	tripartite motif containing 28
TRIM33	H. sapiens	PTC7, RFG7, ECTO, TF1G, TIF1G, TIFGAMMA, TIF1GAMMA	tripartite motif containing 33
TRIM37	H. sapiens	MUL, POB1, TEF3	tripartite motif containing 37
TRIM4	H. sapiens	RNF87	tripartite motif containing 4
TRIM52	H. sapiens	RNF102	tripartite motif containing 52
TTC37	H. sapiens	Ski3, THES, KIAA0372	tetratricopeptide repeat domain 37

TUBB	H. sapiens	M40, TUBB5, TUBB1, CDCBM6, OK/SW-cl.56, DAAP-285E11.4	tubulin, beta class I
TUBG1	H. sapiens	TUBG, GCP-1, CDCBM4, TUBGCP1	tubulin, gamma 1
UACA	H. sapiens	NUCLING	uveal autoantigen with coiled-coil domains and ankyrin repeats
UBE2D2	H. sapiens	UBC4, UBCH4, PUBC1, UBC4/5, UBCH5B, E2(17)KB2	ubiquitin-conjugating enzyme E2D 2
UBE2D3	H. sapiens	UBC4/5, UBCH5C, E2(17)KB3	ubiquitin-conjugating enzyme E2D 3
UBE2E1	H. sapiens	UBCH6	ubiquitin-conjugating enzyme E2E 1
UBE2I	H. sapiens	P18, UBC9, C358B7.1, LA16c-358B7.1	ubiquitin-conjugating enzyme E2I
UBE2N	H. sapiens	UBC13, UbcH13, UbcH-ben, HEL-S-71, UBCHBEN; UBC13	ubiquitin-conjugating enzyme E2N
UBXN6	H. sapiens	UBXD1, UBXDC2	UBX domain protein 6
UNC80	H. sapiens	UNC-80, C2orf21	unc-80 homolog (C. elegans)
VCP	H. sapiens	p97, TERA, ALS14, IBMPFD, HEL-220, IBMPFD1, HEL-S-70	valosin containing protein
VDAC2	H. sapiens	POR, RP11-375G3.1	voltage-dependent anion channel 2
WDYHV1	H. sapiens	C8orf32	WDYHV motif containing 1
XPO1	H. sapiens	emb, CRM1, exp1	exportin 1
YWHAB	H. sapiens	HS1, GW128, YWHAA, KCIP-1, HEL-S-1, RP1-148E22.1	tyrosine 3-monooxygenase/tryptophan 5-monooxygenase activation protein, beta
ZBTB1	H. sapiens	ZNF909	zinc finger and BTB domain containing 1
ZFP82	H. sapiens	ZNF545	ZFP82 zinc finger protein
ZIM2	H. sapiens	ZNF656	zinc finger, imprinted 2
ZNF324B	H. sapiens	-	zinc finger protein 324B
ZNF333	H. sapiens	-	zinc finger protein 333
ZNF418	H. sapiens	-	zinc finger protein 418
ZNF43	H. sapiens	HTF6, KOX27, ZNF39L1	zinc finger protein 43
ZNF44	H. sapiens	ZNF, KOX7, ZNF55, ZNF58, GIOT-2, ZNF504	zinc finger protein 44
ZNF460	H. sapiens	HZF8, ZNF272	zinc finger protein 460
ZNF484	H. sapiens	BA526D8.4, RP11-526D8.9	zinc finger protein 484
ZNF582	H. sapiens	-	zinc finger protein 582
ZNF747	H. sapiens	-	zinc finger protein 747
ZNF764	H. sapiens	-	zinc finger protein 764
ZNF776	H. sapiens	-	zinc finger protein 776
CREBBP	M. musculus	CBP, KAT3A, CBP/p300, p300/CBP, AW558298	CREB binding protein
NFE2L2	M. musculus	Nrf2, AI194320, RP23-374O4.2	nuclear factor, erythroid derived 2, like 2
PARP1	M. musculus	PARP, PPOL, Adprp, ARTD1, parp-1, C80510, Adprt1, sPARP-1, AI893648, 5830444G22Rik	poly (ADP-ribose) polymerase family, member 1
PIAS1	M. musculus	GBP, Ddxbp1, 2900068C24Rik	protein inhibitor of activated STAT 1
POU5F1	M. musculus	Oct4, Oct3, Otf3, Otf4, Otf-3, Otf-4, Oct-3, Oct-4, NF-A3, Otf3g, ...	POU domain, class 5, transcription factor 1

SPI1	M. musculus	Dis1, PU.1, Spi-1, Sfpi1, Dis-1, Sfpi-1, Tcfpu1, Tfpu.1, RP23-20F9.1	spleen focus forming virus (SFFV) proviral integration oncogene
TAL1	M. musculus	Scl, tal-1, bHLHa17, SCL/tal-1, RP23-295C20.6	T cell acute lymphocytic leukemia 1
TFCP2L1	M. musculus	Cp2l1, LBP-9, Crtr-1, AA575098, Tcfcp2l1, 4932442M07Rik, 1810030F05Rik, D930018N21Rik	transcription factor CP2-like 1
TRIM24	M. musculus	TIF1, Tif1a, AI447469, TIF1alpha, TIF1-alpha, D430004I05Rik, A130082H20Rik	tripartite motif-containing 24
ORF3	MERS-CoV	G128_gp03, ORF3_CVEMC, MERS-CoV ORF3, PRO_0000422435	NS3 protein
SUMO3	R. norvegicus	-	small ubiquitin-like modifier 3
SMAD4.1	X. laevis	jip, dpc4, smad4, madh4, smad4a, Xsmad4, xsmad4a, smad4alpha, XSmad4alpha	SMAD family member 4, gene 1
XPO1	X. laevis	Xpo, crm1, exportin-1	exportin 1

1.2 Information of the structures present in the PDB and used in this work.

Table S2. Considered X-ray crystallographic structures.

PDB	Method	Resolution	Gene	ligandID
3O33	X-ray diffraction	2	TRIM24	
3O34	X-ray diffraction	1.9	TRIM24	H3(13-32)K23ac
3O35	X-ray diffraction	1.76	TRIM24	H3(23-31)K27ac
3O36	X-ray diffraction	1.7	TRIM24	H4(14-19)K16ac
3O37	X-ray diffraction	2	TRIM24	H3(1-10)K4
4YAB	X-ray diffraction	1.9	TRIM24	4CN
4YAD	X-ray diffraction	1.73	TRIM24	4A7
4YAT	X-ray diffraction	2.18	TRIM24	4A8
4YAX	X-ray diffraction	2.25	TRIM24	4AE
4YBM	X-ray diffraction	1.46	TRIM24	4BJ
4YBS	X-ray diffraction	1.83	TRIM24	4BK
4YBT	X-ray diffraction	1.82	TRIM24	4BV
4YC9	X-ray diffraction	1.82	TRIM24	4C1
4ZQL	X-ray diffraction	1.79	TRIM24	4QH
5H1T	X-ray diffraction	1.95	TRIM24	7FF
5H1U	X-ray diffraction	1.9	TRIM24	6KT
5H1V	X-ray diffraction	2	TRIM24	7FU
3U5M	X-ray diffraction	3.08	TRIM33α	
3U5N	X-ray diffraction	1.95	TRIM33α	H3(1-28)K9me3K14acK18acK23ac
3U5O	X-ray diffraction	2.7	TRIM33α	H3(1-22)K9me3K14acK18ac
3U5P	X-ray diffraction	2.8	TRIM33α	H3(1-20)K9me3K14ac
5MR8	X-ray diffraction	1.74	TRIM33β	H3K9ac

2RO1	Solution NMR		TRIM28	

1.3 Comparison of the main contact regions of the KAc pocket

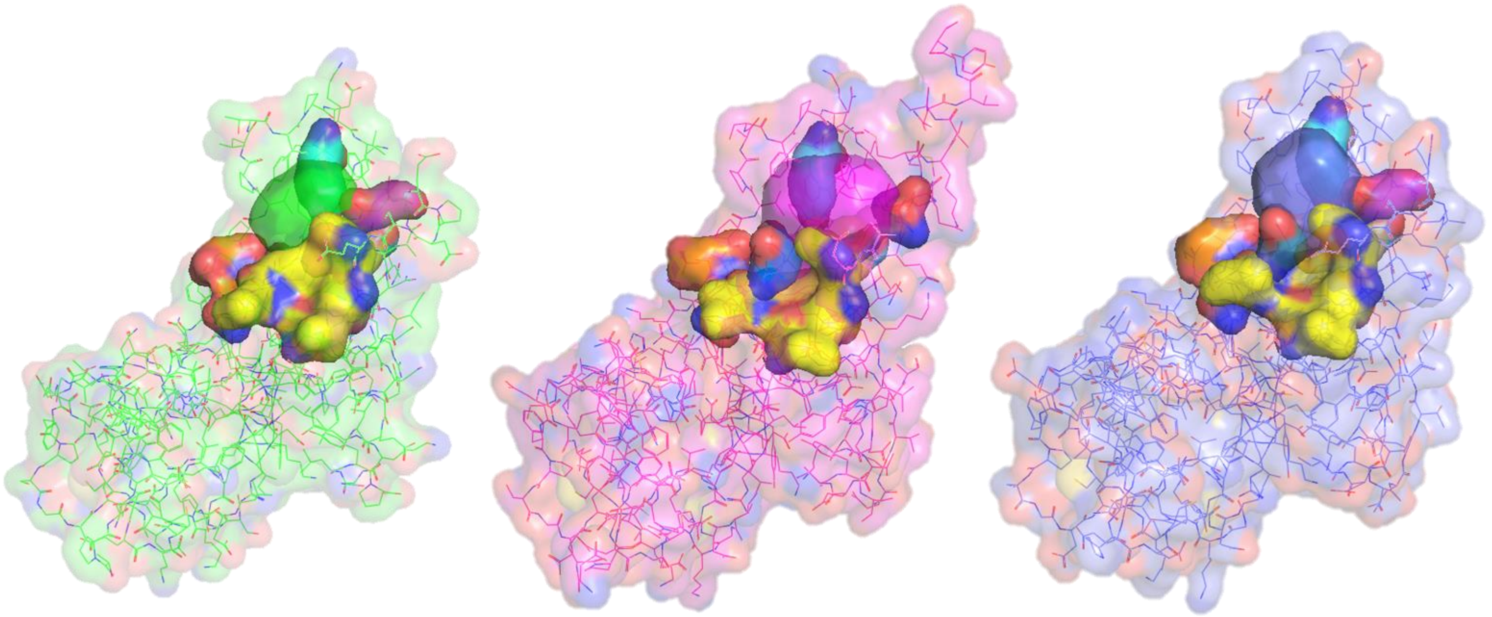

Figure S1. Analysis of the main hydrophobic (yellow surface) and hydrophilic (purple, orange and blue surfaces) contact regions of the KAc binding site in TRIM24 (green – PDB code: 4yc9[42]), TRIM33α (magenta – PDB code: 3u5o[39]) and TRIM33β (violet – PDB code: 5mr8[36]).

1.4 Energetic scanning of residues

Table S3. *Energetic evaluation of residues contribution to the TRIM24 KAc pocket MIFs.*

**TRIM24**					
**Res**	**Chain**	**Number**	**CRY**	**O**	**N1**
**LEU**	**A**	**922**	-10.51		-169.15
**ALA**	**A**	**923**	-77.70	-6.63	-625.91
**PHE**	**A**	**924**	-34.17	-13.31	-9.47
**GLN**	**A**	**925**	-1.69		
**ASP**	**A**	**926**	-33.03	-61.37	-148.82
**PRO**	**A**	**927**	-3.76		-129.02
**VAL**	**A**	**928**	-85.92	-129.30	-23.58
**PRO**	**A**	**929**	-65.73	-4.94	-9.76
**THR**	**A**	**931**	-0.43	-5.42	-38.15
**VAL**	**A**	**932**	-69.64	-1.08	-27.34
**PRO**	**A**	**933**	-3.60	-1.21	-51.04

**TYR**	**A**	**935**	-30.45	-201.67	-80.78
**TYR**	**A**	**936**	-1.33	-24.64	-11.88
**MET**	**A**	**943**	-15.03	-44.10	-305.97
**ASP**	**A**	**944**	-14.66		-50.46
**ILE**	**A**	**972**	-11.69	-1.67	-170.69
**ASN**	**A**	**975**	-7.83	-64.45	-6.25
**CYS**	**A**	**976**	-25.65	-1.09	-29.91
**PHE**	**A**	**979**	-29.20	-1.44	-60.79
**ASN**	**A**	**980**	-105.22	-227.40	-839.72
**GLU**	**A**	**981**	-3.66	-89.56	
**SER**	**A**	**984**	-6.49	-69.38	-77.95
**GLU**	**A**	**985**	-5.27	-2.87	-23.57
**VAL**	**A**	**986**	-64.97	-58.09	-18.02

Table S4. Energetic evaluation of residues contribution to the TRIM33α KAc pocket MIFs.

**TRIM33α**					
**Res**	**Chain**	**Number**	**CRY**	**O**	**N1**
**ILE**	**A**	**980**	-5.00		-32.96
**GLU**	**A**	**981**	-140.15	-65.27	-1171.92
**PHE**	**A**	**982**	-27.11	-12.27	-6.98
**GLN**	**A**	**983**	-1.50		
**GLU**	**A**	**984**	-1.48	-14.74	-54.22
**PRO**	**A**	**985**	-4.53	-2.26	-8.45
**VAL**	**A**	**986**	-56.59	-104.62	-6.95
**PRO**	**A**	**987**	-4.15		-1.06
**SER**	**A**	**989**	-0.45	-8.17	-62.49
**ILE**	**A**	**990**	-71.63	-22.08	-39.92
**PRO**	**A**	**991**	-26.66	-7.69	-135.21
**ASN**	**A**	**992**		-5.35	
**TYR**	**A**	**993**	-33.63	-362.54	-170.77
**TYR**	**A**	**994**	-2.20	-41.18	-2.86
**MET**	**A**	**1001**	-6.91	-29.53	-282.91
**ASP**	**A**	**1002**	-22.59		-145.93
**ILE**	**A**	**1031**	-17.82	-7.40	-136.32
**ASN**	**A**	**1034**	-11.69	-54.18	-1.15
**CYS**	**A**	**1035**	-65.82	-86.75	-285.47
**GLU**	**A**	**1036**	-1.11		-14.29
**PHE**	**A**	**1038**	-79.39	-38.14	-66.84
**ASN**	**A**	**1039**	-80.27	-72.57	-503.23
**GLU**	**A**	**1040**			
**MET**	**A**	**1042**	-80.10	-59.18	-199.60
**LYS**	**A**	**1043**	-14.95	-19.95	
**GLN**	**A**	**1046**	-57.31	-23.22	-41.18
**SER**	**A**	**1060**	-56.17	-371.38	-339.30
**GLU**	**A**	**1061**	-62.58	-8.10	-614.18

**VAL**	**A**	**1062**	-65.98	-16.39	-33.14
**ALA**	**A**	**1063**	-8.951	-4.34	-14.452
**LYS**	**A**	**1067**		-1.622	

Table S5. Energetic evaluation of residues contribution to the TRIM33β KAc pocket MIFs.

**TRIM33β**					
**Res**	**Chain**	**Number**	**CRY_max (kcal/mol)**	**O_max (Kcal/mol)**	**N1_max (kcal/mol)**
**ILE**	**A**	**980**	-2.01	-1.14	-135.06
**GLU**	**A**	**981**	-165.22	-70.23	-1388.19
**PHE**	**A**	**982**	-33.31	-19.15	-6.99
**GLN**	**A**	**983**	-0.38		
**GLU**	**A**	**984**	-19.24	-69.07	-61.83
**PRO**	**A**	**985**	-2.75	-2.69	-97.22
**VAL**	**A**	**986**	-63.33	-114.49	-11.06
**PRO**	**A**	**987**	-22.84	-7.45	
**SER**	**A**	**989**	-0.28	-4.36	-21.17
**ILE**	**A**	**990**	-78.20	-26.11	-20.58
**PRO**	**A**	**991**	-4.07	-1.03	-68.60
**TYR**	**A**	**993**	-22.19	-256.96	-83.95
**TYR**	**A**	**994**	-1.66	-13.72	
**MET**	**A**	**1001**	-13.85	-64.28	-290.17
**ASP**	**A**	**1002**	-22.17	-1.84	-96.93
**ILE**	**A**	**1031**	-12.81	-3.53	-168.77
**ASN**	**A**	**1034**	-6.56	-40.47	-3.55
**CYS**	**A**	**1035**	-27.98		-27.67
**PHE**	**A**	**1038**	-27.13	-12.94	-67.81
**ASN**	**A**	**1039**	-101.72	-265.87	-802.33
**GLU**	**A**	**1040**	-28.20	-112.00	-77.80
**SER**	**A**	**1043**	-7.25	-74.49	-64.86
**GLU**	**A**	**1044**	-12.56	-8.31	-10.11
**VAL**	**A**	**1045**	-46.72	-109.72	-28.72

1.5 Consideration about the X-ray conserved water molecule

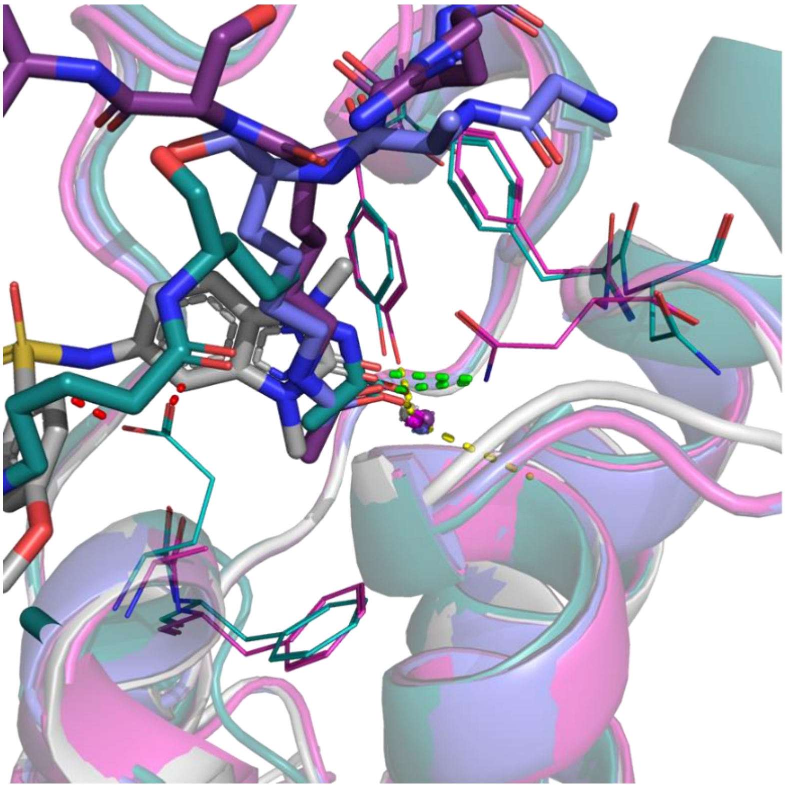

Figure S2. Structural comparison of TRIM24 and TRIM33α. TRIM24 in its free-ligand state (PDB code: 3o33[57] in magenta) and TRIM24 in complex with: ligand 4A8 (PDB code: 4yat[42] in grey), H3(23-31)K27ac peptide (PDB code: 3o35[57] in violet) and H4(14-19)K16ac peptide (PDB code: 3o36[57] in purple). TRIM33α in complex with H3(1-20)K9me3K14ac peptide (PDB code; 3u5p[39] in teal). The X-ray Ligand 4A8 and the other 3 peptides reported have a similar binding mode, in particular the position of the carbonyl group is always the same (suggesting that also the interaction with the protein is the same). One of these peptides is co-crystallized with TRIM33α (coloured in teal) and also that one conserved the binding mode despite the different orientation of the Asparagine residue. Observing the majority of the known TRIM24 structures (both Apo and Holo), there is always a conserved and structural water molecule in the bottom of the pocket (close to the asparagine residue).

1.6 Docking in TRIM33α without the conserved water molecule

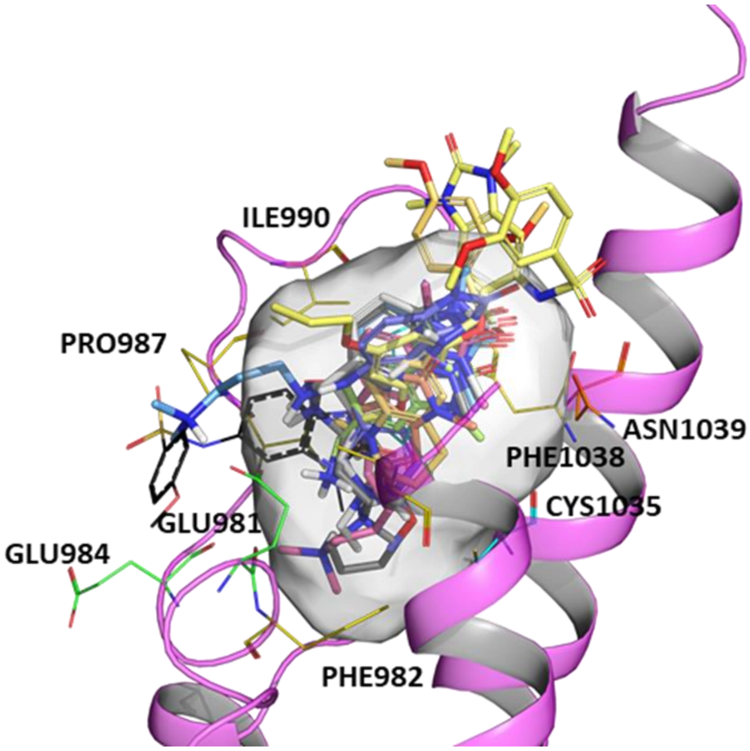

Figure S3. The obtained wrong docking poses in BRD of TRIM33α (PDB code: 3u5o[39], represented in magenta) using GLIDE without water constraint are reported. Reference X-ray ligand 4A8 (compound 3) for its binding mode (represented as black wire). The main interacting pocket residues are highlighted: lipophilic residue as yellow wires, ASN1039 as orange wires, TYR993 and CYS1035 as cyan wires and GLU981 and GLU984 as green wires. Compounds 2, 3, 4, 5, 6, 7, 8, 9 and 10 are represented as sticks in multiple colours.

1.7 Docking in TRIM33β

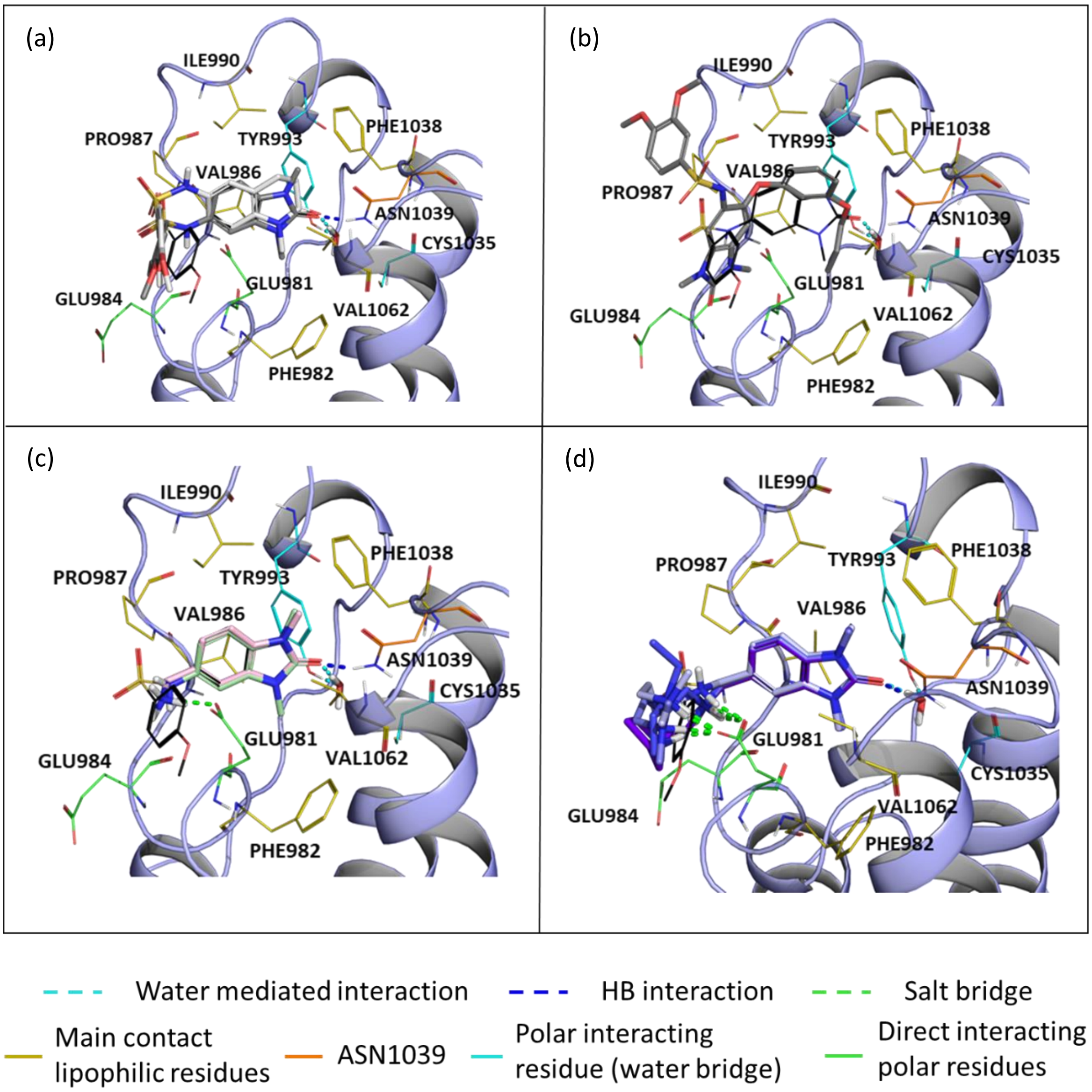

Figure S4. The obtained docking poses in BRD of TRIM33β (PDB code: 5mr8[36], represented in purple) using GLIDE are reported. Reference X-ray 4A8 ligand for its binding mode (represented as black wire). The main interacting pocket residues are highlighted: lipophilic residue as yellow wires, ASN1039 as orange wires, TYR993 and CYS1035 as cyan wires and GLU981 and GLU984 as green wires. Moreover, the water mediated interaction as represented in cyan, the direct HB interaction in blue and the salt bridge interactions in green. (a) Representation of compounds 2 (as light grey stick) and 3 (as grey stick). (b) Representation of compound 4 (as dark grey stick). (c) Representation of scaffold 5 (as pale green stick) and 6 (as light pink stick). (d) Representation of compound 7 (as light blue stick), 8 (as tv blue stick), 9 (as slate stick) and 10 (as purple stick) in their first possible conformation.

1.8 Comparison between the canonical BRD of TRIM24 and the not-canonical BRD of TRIM28

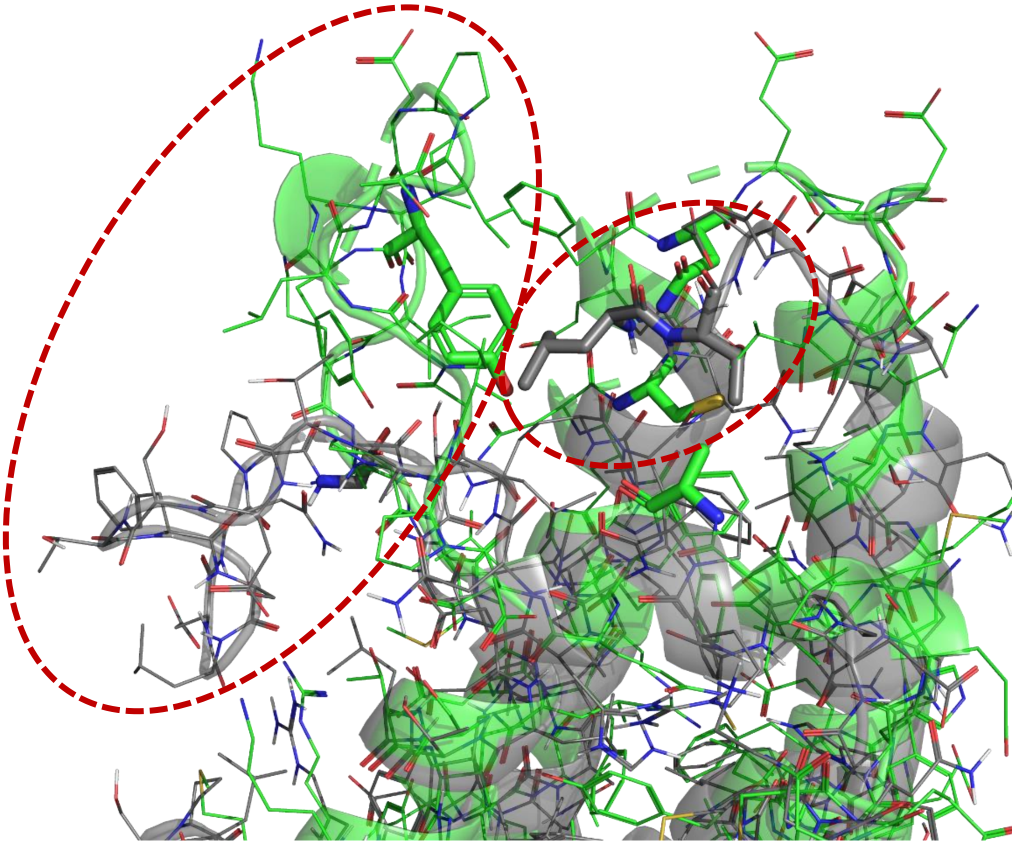

Figure S5. Overlap of TRIM24 structure in green (PDB code: 4yat[42]) and aligned TRIM28 structure in grey (PDB code: 2ro1[60]). The main residues of the KAc binding site of the BRD domain are represented as sticks to highlight the main differences and the different site conformation.

1.9 K_d determination of the Peptide/TRIM systems

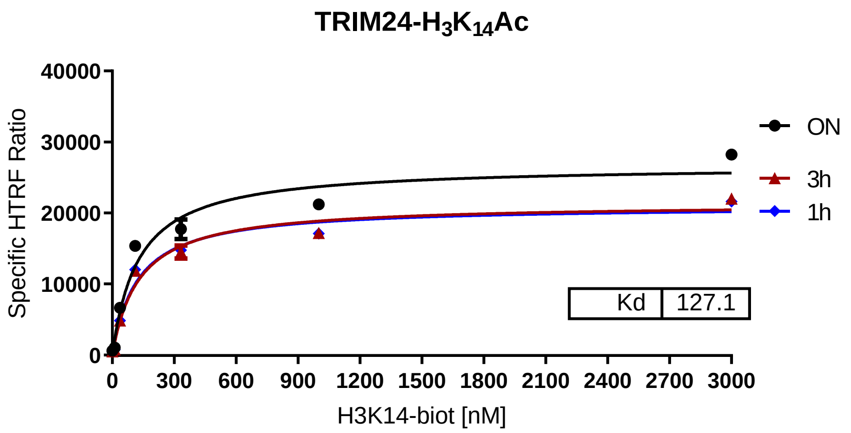

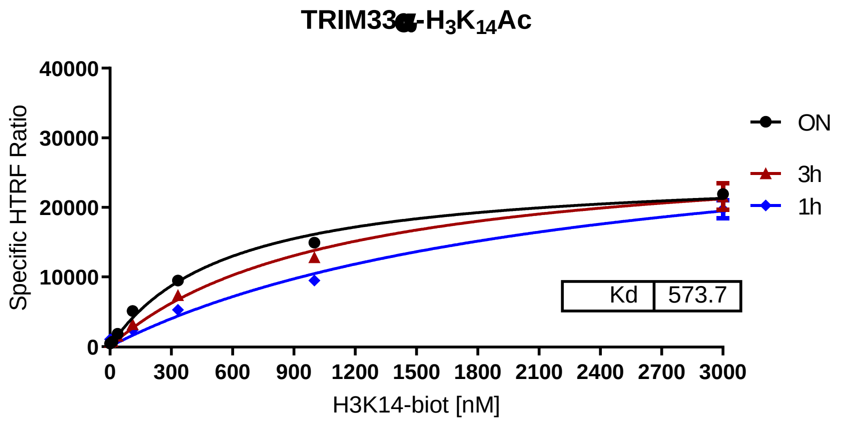

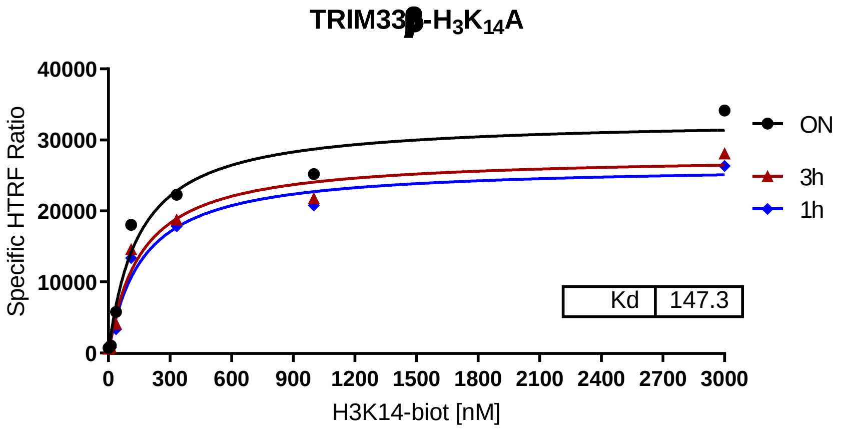

Figure S6. HTRF assay – titration curves for K_d detemination of Biotinylated Histone $H_3K_{14}Ac$ peptide with TRIM24, TRIM33α and TRIM33β.

1.10 Possible chain conformations from docking analysis

In the obtained X-ray crystallographic complexes of TRIM33α and compounds 8, 9 and 10 the density map of the chain was not detected due to the not optimal resolution of the structures and its high degree of movement. In fact, the chain is highly exposed to the solvent and as highlighted also by the docking analysis, for each compound several poses were obtained, with comparable docking score, just differentiated by the position of the chain. This is free to move and rotate and may interact both with the GLU981 and GLU984. Just the position of this chain can also influence the position of some flexible residues close to the binding site. The docking analysis was performed using standard parameters imposing the interaction with the crystallographic water stored in the input receptor and without any optimization step. However, considering the different conformers obtained and the possible influence of the different chain positions on the flexible residues of the protein, the obtained docking poses were post processed using the Refine Protein-Ligand Complex tool in Maestro. This is a refinement step performed in order to relax the complex and to minimise the interactions and the relative atoms positions. It is evident that the chain is actually free to move by folding and interacting with a double anchor on GLU981 or to extend and interact with GLU984 and GLU983 on the backbone. Precisely for this reason and considering the non-optimal resolutions of the structures obtained, the position of the chain is not defined and the chain was deleted from the deposited PDB structures of TRIM33α.

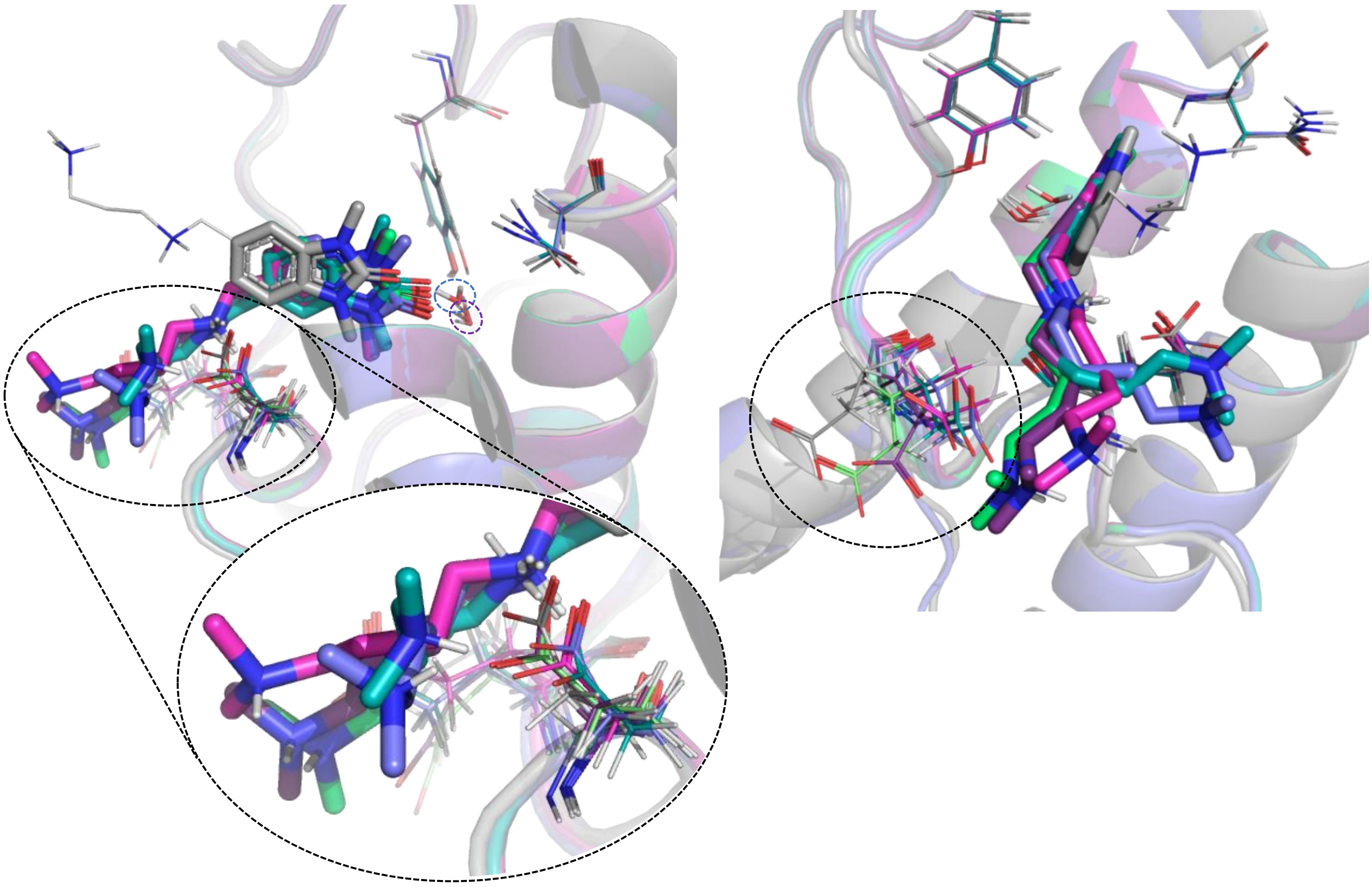

Figure S7. Obtained X-ray structure of TRIM33α co-crystallized with compound 8 (in grey) and its obtained docking poses (multi-colours – PDB code: 3u5o[39] as input receptor for the analysis) are represented. The resolved scaffold and docked ligands are represented as sticks while the crystallographic ligand chain (unsolved) is represented as wire. X-ray conserved water is well defined and highlighted by the light blue circle, while the water molecule used in the docking analysis in shown by the purple circle. Moreover, the boxes show how the various conformation of the chain, in the different docking poses, influences the position of the residues involved in the interactions.

1.11 TRIM33α ligands: Experimental binding mode vs Predicted pose

In Figure below, the comparison between the predicted and the experimental binding mode is reported. The obtained X-ray crystallographic structures of TRIM33α in complex with ligands 8, 9 and 10 confirm both the binding of the ligands into the KAc region and the predicted binding mode. The X-ray scaffolds of ligands 8, 9 and 10 are overlapped with the docking pose previously reported. Moreover, also the conserved water molecule is detected in the X-ray structure validating the water considerations made and its key role in the binding to TRIM33α. Focusing on the scaffold of the ligands, there is a little shift of the x-ray structure, particularly with ligand 8 (Figure 8a). However, the water mediated network between the carbonyl group of the ligands and the TYR993 and CYS1035 is still conserved both in terms of interaction made and also in terms of bond distances between the parts (Lig-Wat from 3.0 Å to 3.2 Å, THR993-Wat from 2.5 Å to 2.7 Å and CYS1035-Wat from 3.1 Å to 3.3 Å). In fact, the water molecule and the pocket residues are also shifted and the geometry of the interactions is always preserved.

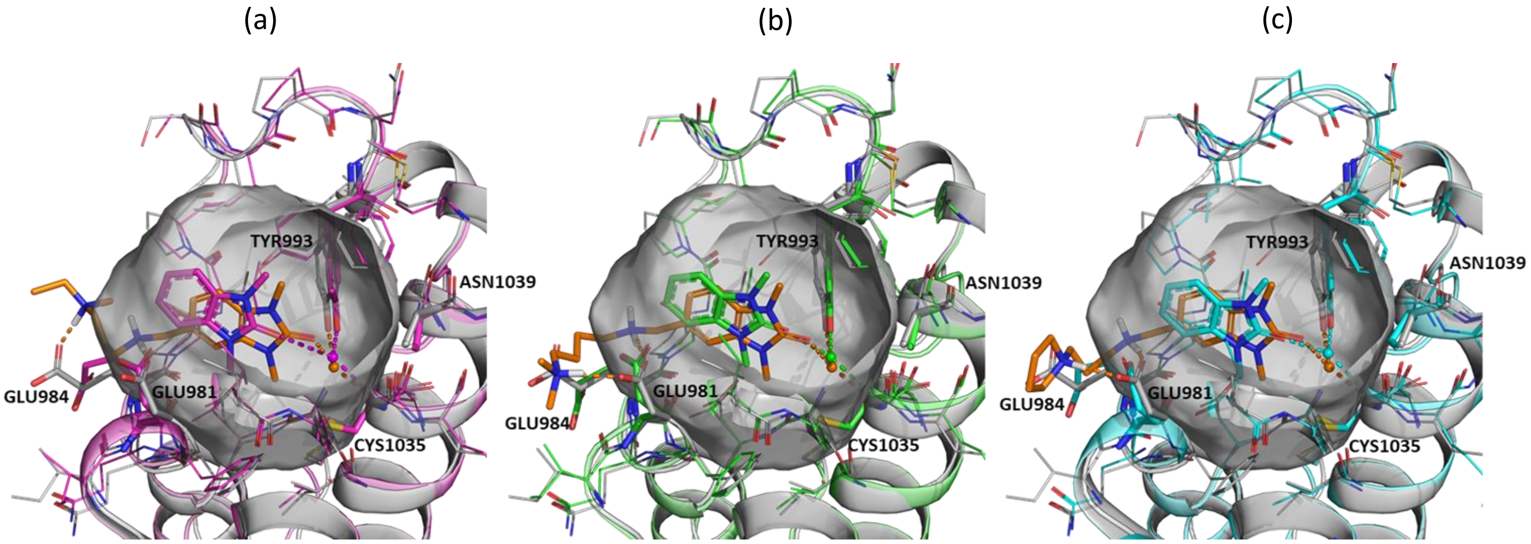

Figure S8. Overlap between the predicted docking poses (PDB code: 3u5o[39] in grey) and the obtained X-ray crystallographic structures (TRIM33 protein and residues coloured according to the ligand). KAc binding site view of TRIM33α PHD-BRD with (a) 8 (in sticks, X-ray pose in magenta and docking pose in orange), (b) 9 (in sticks, X-ray pose in green and docking pose in orange), (c) 10 (in stick, X-ray pose in cyan and docking pose in orange). Water X-ray molecules and the one used in the docking are displayed as spheres coloured according with the related ligand. Hydrogen bonds are represented as dashed lines coloured according with the related ligand. Oxygen and nitrogen atoms are coloured red and blue, respectively.

Experimental section

2.1 HTRF assay

HTRF reagents

EPIgeneous™ Binding Domanin Discovery Kit: all reagents were obtained from PerkinElmer|cisbio (Codolet, FR)

- Binding Domain Diluent Buffer (Diluent - catalog number 62DLBDDF)
- Binding Domain Detection Buffer #1 (Buffer1 - catalog number 62DB1FDG)
- Binding Domain Detection Buffer #2 (Buffer2 - catalog number 62DB2FDG)
- Strepatavidin-d2 Reagent (S-D2 - catalog number 610SADLA)
- Streptavidin-XL665 Reagent (S-XL665 - catalog number 610SAXLA)
- Anti GST-Eu Cryptate Antibody (αGST Eu - catalog number 61GSTKLA)
- Anti GST-Tb Cryptate Antibody (αGST Tb - catalog number 61GSTTLA)

HTRF® detection reagents were resuspended and stored according to manufacturer's recommendations.

Proteins, peptides and ligands:

- Human recombinant TRIM24 bromodomain (residues 824-1014) expressed as an N-terminal GST-TEV-fusion protein in E. coli (Reaction Biology corp., Malvern, USA; catalog number RD-11-186)
- Human recombinant TRIM33α PHD-bromodomain (residues 885-1091) expressed as an N-terminal GST-fusion protein in E. coli (Reaction Biology corp., Malvern, USA; catalog number RD-11-238)
- Human recombinant TRIM33β bromodomain construct (residues 885-1074) expressed as an N-terminal GST-fusion protein in E. coli (Reaction Biology corp., Malvern, USA; catalog number RD-11-290)

The proteins were initially stored at -80 °C in 1 μL aliquots. When necessary, to the aliquots were added the volume of Binding Domain Diluent Buffer suitable to reach the desired concentration for the assay.

- Histone $H_3K_{23}Ac$ peptide, Biotinylated (Creative Biomart Epigenetics, Milan, IT; catalog number SP-0367)
- Histone $H_4K_{5,8,12,16}Ac$ peptide, Biotinylated (Creative Biomart Epigenetics, Milan, IT; catalog number SP-0238)
- Histone $H_3K_{14}Ac$ peptide, Biotinylated (Creative Biomart Epigenetics, Milan, IT; catalog number SP-0367).

All peptides were initially suspended into Binding Domain Diluent Buffer to obtained stock solution at concentration 350 μM and stored at -20 °C in 1 μL aliquots. When necessary, to the aliquots were added the volume of Binding Domain Diluent Buffer suitable to reach the desired concentration for the assay.

- Two of the tested ligands (4A7 and 4A8) were acquired (Mcule, Hungary) while the others were synthesized by the research group (See Chemistry section below)

All compounds were initially dissolved into DMSO and stored at -20 °C in 1 μL aliquots. When necessary, to the aliquots were added the volume of Binding Domain Diluent Buffer suitable to reach the desired concentration for the assay.

Filter set:

The reader must be appropriately configured for HTRF readout by setting up the measurement conditions in the Gen5 Reader Control and data Analysis Software (see Table below). HTRF assay must be read using the filter-based detection mode only.

Settings	Filter set 1	Filter set 2
Excitation wavelength<br>Emission wavelength<br>Dichroic mirror	330/80<br>620/10<br>365	330/80<br>665/10<br>365
Gain	Autoscale	
Read speed	Normal<br>Delay after plate movement: 100 msec<br>Measurement per data point: 10<br>Lamp energy: High (more sensitivity)	
Time resolved	Delay time: 150 μsec<br>Data collection time: 500 μsec	

Kit and peptide selection procedure:

In order to identify the optimal peptide and optimal kit for each TRIM, a preliminary HTRF binding assay was performed using for each TRIM all the three bought peptides in combination with three different EPIgeneous™ Binding Domanin Discovery Kit (Kit-A, Kit-B, Kit-C). All Kits comprise the same Diluent but differ for the combination of the other components:

- Kit-A: Anti GST-Eu Cryptate Antibody & Strepatavidin-d2 Reagent (Buffer1)
- Kit-B: Anti GST-Eu Cryptate Antibody & Streptavidin-XL665 Reagent (Buffer1)
- Kit-C: Anti GST-Tb Cryptate Antibody & Streptavidin-XL665 Reagent (Buffer2)

HTRF assays were performed in white 384 Well Small Volume™ HiBase Polystyrene Microplates (Greiner) with a total working volume of 20 μL. TRIMs aliquots were diluted in Diluent to get a 25 nM working solutions (5X, the final concentration in the total well volume, 20 μL, should be 5 nM) and 4 μL were dispensed in each well. Similarly, the peptides aliquots were suitably diluted in Diluent to get 15 μM working solutions (5X, the final concentration in the total well volume, 20 μL, should be 3 μM) and 4 μL were dispensed in each well. Then, the Streptavidin was diluted in the opportune Buffer, 1 or 2 according to the kit indications, to get 1500 nM working solutions (4X, the final concentration in the total well volume, 20 μL, should be 0,375 μM, 1/8 of the Peptide concentration) and 5 μL were dispensed in each well. Finally, the αGST aliquots (50X) were diluted 50-fold in appropriate buffer, 1 or 2 according to the kit indications, to get the working solution and 5 μL were dispensed in each well.
The GST protein is missing in the control wells and, in substitution, 4 μL of Diluent (in which the protein is dissolved) were added to reach the overall assay volume. Each measurement was performed in triplicate.

To explore the stability and reliability of HTRF assay, repeated tests for each kit were performed and the Z′ value was calculated. The Z′-factor is often used as a performance indicator for which an excess of 0.5 indicates high assay performance.

Peptide titration procedure:

TRIMs concentration is fixed at 5 nM final concentration (see previously section). Peptide stock solutions was diluted by Diluent Buffer to prepare the 5x working solutions (5X, the final max concentration in the total well volume, 20 μL, should be 3 μM) and then underwent six serial three-fold dilutions. The tested range of peptide concentration was from 3000 nM to 4 nM. As the biotin/streptavidin molar ratio must be 8/1 during the detection step, Streptavidin was diluted in the opportune Buffer 1 or 2 according to the supplier indications, to get 4X working solutions (4X, the final max concentration in the total well volume, 20 μL, should be 0,375 μM) and then serially diluted 1:3. Finally, the αGST aliquots (50X) were diluted 50-fold in the appropriate buffer, 1 or 2 according to the kit indications, to get the working solution and 5 μL were dispensed in each well.

For each peptide-biotin concentration a negative control is performed by not adding the GST protein to the wells, replacing it with the same volume of Diluent. Each measurement was performed in triplicate.

Competition binding procedure:

The best assay conditions were determined in the previous steps (kit and peptide identification and peptide-titration). TRIM concentration was fixed at 5 nM final concentration (see Kit and peptide selection procedure section). The compound stock solutions were diluted in Diluent to get 200 μM working solution (10X, the final max concentration in the total well volume, 20 μL, should be 20 μM) and then serially diluted 1:2. The tested range of compound concentration was from 20 μM to 2.5 μM. Peptide concentration was fixed for each TRIM at the relative Kd value previously determined, in order to compare different systems (the peptide in fact has different affinities for the TRIMs, but with a peptide concentration equal to Kd, regardless of the affinities, only half of the TRIMs will be occupied at equilibrium). Peptides stock solutions were diluted in Diluent Buffer to get a 5X working solution depending on the final optimal concentration (Kd concentration). Streptavidin and αGST were prepared as reported in the peptide titration procedure. In addition to the usual negative control, a positive control, in which there are all the reagents except the compound, was added.

HTRF data analysis:

- As regards the Kit and peptide selection and the peptide titration, the data analysis is the same:
    1. Per each well (both positive signal and negative controls) the ratio between the fluorescence intensities were calculated:

$$\text{Ratio}_i = \frac{Fluorescence\ Intensity\ 665\ nM_i}{Fluorescence\ Intensity\ 620\ nM_i} \times 10000^*$$

*The 10^4 multiplying factor is introduced for easier the data processing

    2. The triplicate ratios were averaged:

$$\underline{Ratio_i} = \frac{Ratio_{i,1} + Ratio_{i,2} + Ratio_{i,3}}{3}$$

    3. Comparing each mean ratio of the positive signal wells with the control ones, the S/B ratio is determined:

$$\text{S/B (\%)} = \frac{\underline{Ratio}\ (Positive\ signal)_i}{\underline{Ratio}\ (Negative\ control)} \times 100$$

4. Z′ calculation needs four variables: the average signal of the positive controls; the average signal of the negative controls; the standard deviation of the positive controls; the standard deviation of the low controls. The value is calculated as follows:

$$Z' = 1 - \frac{(3SD_{+} + 3SD_{-})}{|AVE_{+} - AVE_{-}|}$$

- As regards the competition binding assay, the data analysis involves additional steps. In order to compare different experiments (for example the same compound in different TRIMs or multiple compounds in the same TRIM), since these are not perfectly reproducible, it is necessary to normalise the data. After calculating the ratio (665/620), to reduce the variability between wells and reduce the interference effect of each possible compound to the fluorescence measurement, the data are normalised for ΔF:

   1. The ratio must be calculated for each well individually. The mean and standard deviation can then be worked out form replicates.

$$\text{Ratio}_i = \frac{Fluorescence\ Intensity\ 665\ nM_i}{Fluorescence\ Intensity\ 620\ nM_i} \times 10000^{*}$$

*The 10^4 multiplying factor is introduced for easier the data processing

   2. ΔF is used for the comparison of day-to-day runs of the same assay. It reflects the signal to background of the assay. The negative control plays the role of an internal assay control.

$$\Delta F = \frac{Ratio_i - Ratio_{(Negative\ controls)}}{Ratio_{(Negative\ controls)}} \% \ \text{->}\ \frac{SIGNAL-BACKGRUOND}{BACKGROUND}$$

   3. To compare different competitive assays, the data are normalized using the ΔF_{max}

$$\frac{\Delta F_i}{\Delta F_{max}} \% \ \text{->}\ \Delta F_{max} = \frac{Ratio_{(Positive\ control)} - Ratio_{(Negative\ controls)}}{Ratio_{(Negative\ controls)}} \%$$

Delta F max corresponds to 100% of the HTRF signal. This occurs when the compound is not present in solution and the fluorescence transfer is maximum due to the bind between peptide and protein. In presence of the compound, if this competes with the peptide for binding to the protein, the detected signal will be lower because the fluorescence transferred from the donor (αGST – bind to the protein) to the acceptor (Streptavidin - bind to the peptide) will be lower. To explore the stability and reliability of high-throughput screening based on the HTRF assay, repeated tests were performed at a single concentration of antibody reagents. Then the Z value and the S/B value were calculated ($Z = 1-(3 *(\sigma p + \sigma n)/|(\mu p - \mu n)|)$)

Data manipulation and calculations were performed with Microsoft Excel. All curve-fitting operations were performed using GraphPad Prism Version 8.4.3. The saturation binding experiments to determine the Kd value of each TRIM-peptide system were analysed by curve fitting using the 1-site specific binding function.

2.2 Chemistry

General Synthetic Chemistry Methods

Unless otherwise noted, starting materials, reagents, and solvents were purchased from commercial suppliers and were used as received without further purification.

Reactions were routinely monitored by thin-layer chromatography (TLC) performed on silica gel 60 F254 (layer 0.2 mm) pre-coated aluminium foil (with fluorescent indicator UV254) (Sigma-Aldrich). Developed plates were air-dried and visualized by UV detector (λ: 254/365 nm) and/or by staining and warming with potassium permanganate or ninhydrin. Flash column chromatography was performed on Merck silica gel 60 (mesh 230-400). Automated flash chromatographic purifications were performed using Biotage® Selekt (Cartridge: Sfär Silica HC Duo 5g or 10g). ^{1}H NMR and ^{13}C NMR spectra were recorded at room temperature at 400 and 101 MHz, respectively, on a Bruker Avance 400 spectrometer in the indicated solvent by using residual solvent peak as internal standard. Chemical shifts are reported in ppm (δ) and the coupling constants (*J*) are given in Hertz (Hz). Peak multiplicities are abbreviated as follows: s (singlet), bs (broad singlet), d (doublet), dd (double doublet), t (triplet), dt (double triplet), q (quartet), p (pentet), and m (multiplet). High-Resolution Mass Spectroscopy (HRMS) analyses were carried out on Agilent Technologies 6540 UHD Accurate Mass Q-TOF LC-MS system. The purity of all synthesized compounds was confirmed to be >95% by UPLC-MS. The analyses were carried out according to the method listed below. The mobile phase was a mixture of water (solvent A) and acetonitrile (solvent B), both containing formic acid at 0.1%. Method: Phenomenex Luna Omega C18 Polar, 1.6 μm (C18, 100 x 2.1 mm) column at 40° C using a flow rate of 0.65 mL/min in a 10 min gradient elution. Gradient elution was as follows: 99.5:0.5 (A/B) to 5:95 (A/B) over 8 min, 5:95 (A/B) for 2 min, and then reversion back to 99.5:0.5 (A/B) over 0.1 min. The UV detection is an averaged signal from a wavelength of 190 nm to 640 nm and mass spectra are recorded on a mass spectrometer using positive mode electro spray ionization.

Compounds 1,3-dimethyl-2-oxo-2,3-dihydro-1*H*-benzo[*d*]imidazole-5-carbaldehyde, 3,4-diaminobenzonitrile, 5-nitro-1*H*-benzo[*d*]imidazol-2(3*H*)-one and resorcinol were purchased from Fluorochem.

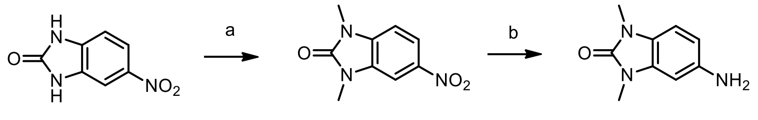

Reagents and conditions: (a) i. 60% NaH, dry DMF, 0 °C-rt, 30 min; ii. CH_3I, dry DMF, rt, 30 min; b) Pd/C, P_{H2} = 1 bar, dry THF:EtOH mixture (1:1), rt, 5 h.

1,3-Dimethyl-5-nitro-1*H*-benzo[*d*]imidazol-2(3*H*)-one (S11).[61]

Under N_2 atmosphere, the solution of 5-nitro-1*H*-benzo[*d*]imidazol-2(3*H*)-one (2.0 g, 11.165 mmol) in dry DMF (12.0 mL) was added to a suspension of NaH 60% (0.937 g, 23.447 mmol) in dry DMF (5.0 mL) and the mixture was stirred at 0° C for 30 minutes. Thus, iodomethane (5.547 g, 39.078 mmol) was added dropwise and the reaction mixture stirred at room temperature for 30 min. The reaction mixture was quenched with EA (10 mL) yielding a precipitate which was filtered and washed with H_2O (10 mL x 3) and then DEE (10 mL x 3). The organic phase recovered from the previous filtration process, added of H_2O (30 mL) and acidified with HCl 2N, was subsequently extracted with EA (10 mL x 5). The reunited organic phases were washed with brine (10 mL x 2), dried over Na_2SO_4, and then evaporated via rotary evaporation. The solids obtained by both filtration and extraction processes were collected together and dried by vacuum to obtain **S11** as a yellow solid (2.2 g, 90% yield). **^{1}H NMR** (400 MHz, $CDCl_3$) δ 8.12 (dd, *J* = 8.6, 2.2 Hz, 1H), 7.89 (d, *J* = 2.2 Hz, 1H), 7.02 (d, *J* = 8.7 Hz, 1H), 3.50 (s, 3H), 3.49 (s, 3H).

5-Amino-1,3-dimethyl-1*H*-benzo[*d*]imidazol-2(3*H*)-one (5).[61]
Under N_2 atmosphere, to the solution of **S11** (2.07g, 10 mmol) in a mixture of THF dry/EtOH dry 2:1 (105 mL) Pd/C 10 % (0.207g) was added and the mixture was stirred at room temperature under H_2 (1 bar) atmosphere for 5 h. Then, the reaction mixture was filtered over a pad of Celite, washed with THF (5.0 mL x 3) and evaporated via rotary evaporation obtaining **5** as a yellow solid (1.760 g, 99% Yield) and used without further purifications. **^{1}H NMR** (400 MHz, DMSO-d_6) δ 6.79 (d, *J* = 8.2 Hz, 1H), 6.36 (d, *J* = 2.0 Hz, 1H), 6.31 (dd, *J* = 8.2, 2.1 Hz, 1H), 4.79 (bs, 2H), 3.22 (s, 3H), 3.21 (s, 3H). **^{13}C NMR** (101 MHz, DMSO-d_6) δ 154.32, 144.31, 130.97, 121.21, 108.43, 107.36, 94.85, 27.26, 27.14. **HRMS** (ESI) *m*/*z* [M + H]+ calcd for $C_9H_{11}N_3O$ 178.09804, found 178.09750. **UPLC** retention time: 1.176 min.

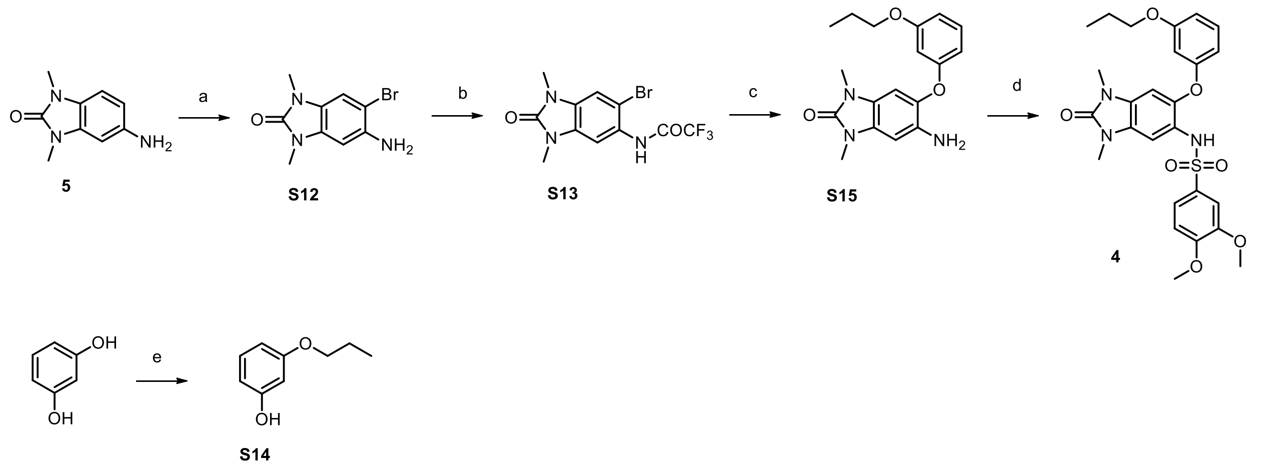

Reagents and conditions: (a) Br_2, AcOH, DCM, 0 °C-rt, 45 min; (b) Trifluoroacetic Anhydride, Et_3N, DMAP, DCM, 0 °C-rt, 5 h; (c) appropriated phenol intermediates (**S14**), CuI, Cs_2CO_3, *N*,*N*-dimethylglycine, dry dioxane, 80°C, overnight; (d) 3,4-dimethoxybenzene-1-sulfonyl chloride, dry Pyr, dry DCM, 2-3 h; (e) K_2CO_3, DMF, 70°C, overnight.

5-Amino-6-bromo-1,3-dimethyl-1*H*-benzo[*d*]28midazole-2(3*H*)-one (S12).[42]
To a solution of **5** (0.429 g, 2.421 mmol) in DCM/AcOH 1:1 (24 mL) at 0°C Br_2 (0.387 g, 2.421 mmol) was added carefully dropwise. After stirring at room temperature for 45 minutes, the reaction mixture was diluted with H_2O (100 mL), neutralized with $NaHCO_3$ aq. sat. (150 mL), and extracted with DCM (50 mL x 5). The reunited organic phases were washed with H_2O (40 mL x 3), brine (20 mL x 2), dried over Na_2SO_4 and evaporated to dryness. The crude was then purified by flash column chromatography on SiO_2 (EA 100%), obtaining **S12** as a yellow solid (0.450 g, 73 % Yield). ^{1}H NMR (400 MHz, $CDCl_3$) δ 7.01 (s, 1H), 6.48 (s, 1H), 3.34 (s, 3H), 3.33 (s, 3H). **^{13}C NMR** (101 MHz, $CDCl_3$) δ 154.58, 138.82, 130.72, 123.78, 110.99, 101.35, 95.68, 27.24, 27.17.

***N*-(6-Bromo-1,3-dimethyl-2-oxo-2,3-dihydro-1*H*-benzo[*d*]imidazol-5-yl)-2,2,2-trifluoroacetamide (S13).**[42]
At 0°C to a solution of **S12** (2.0 g, 7.809 mmol) in DCM (125 mL) DMAP (0.095g, 0.781 mmol), Et_3N (1.581 g, 15.619 mmol), and then dropwise and with vigorous stirring trifluoroacetic anhydride (2.460 g, 11.714 mmol) were added and the reaction mixture was stirred for 2 h by warming up gradually to room temperature. The mixture was quenched with H_2O (20 mL), washed with brine (15 mL x 2), dried over Na_2SO_4 and evaporated to dryness, yielding **S13** as a yellow solid (0.655g, 95 % Yield) after a purification via flash chromatography on SiO_2 (EA, 100%)**^{1}H NMR** (400 MHz, $CDCl_3$) δ 8.43 (s, 1H), 7.99 (s, 1H), 7.17 (s, 1H), 3.42 (s, 3H), 3.40 (s, 3H).

5-Amino-1,3-dimethyl-6-(3-propoxyphenoxy)-1*H*-benzo[*d*]imidazol-2(3*H*)-one (S15).[42]
Under N_2, to the solution of the phenol intermediate **S14** (1.29 g, 8.52 mmol) in dry dioxane (42 mL) **S13** (2.0 g, 5.68 mmol), Cs_2CO_3 (5.74 g, 17.61 mmol), CuI (0.325 g, 1.704 mmol), and *N*,*N*-dimethylglycine (0.586 g, 5.68 mmol) were added and the mixture was stirred at 80°C for 8 h. After TLC showed partial consumption of the starting materials was added dry MeOH (21 mL) (to facilitate the deprotection of the trifluoroacetamide intermediate) and the mixture further stirred at 80°C overnight. The solvent was evaporated to dryness and the crude was purified by flash column chromatography on SiO_2 (EA, 100%). The titled compound was

obtained as a yellow solid (1.159 g, 41 % Yield). ^{1}**H NMR** (400 MHz, $CDCl_3$) δ 7.17 (t, *J* = 8.5 Hz, 1H), 6.63 – 6.55 (m, 3H), 6.56 – 6.48 (m, 2H), 3.86 (t, *J* = 6.6 Hz, 2H), 3.36 (s, 3H), 3.30 (s, 3H), 1.77 (m, *J* = 7.3 Hz, 2H), 1.01 (t, *J* = 7.4 Hz, 3H). ^{13}**C NMR** (101 MHz, $CDCl_3$) δ 160.60, 159.30, 154.84, 138.05, 132.42, 130.15, 129.94, 127.44, 123.43, 108.49, 103.28, 101.48, 97.06, 69.60, 27.28, 27.22, 22.53, 10.53.

***N*-(1,3-Dimethyl-2-oxo-6-(3-propoxyphenoxy)-2,3-dihydro-1*H*-benzo[*d*]imidazol-5-yl)-3,4-dimethoxybenzenesulfonamide (4)**.[42]

To the solution of **S15** (0.040 g, 0.122 mmol) in dry DCM (2 mL) 3,4-dimethoxybenzene-1-sulfonyl chloride (0.043 g, 0.183 mmol) and pyridine (0.0193 g, 0.244 mmol) were added and the reaction mixture was left at room temperature for 3 h. Then, the mixture was diluted with H_2O, acidified with HCl 2N, and extracted with EA (3x 10 mL). Subsequently, the reunited organic phases were washed with brine (2x15 mL) and dried over Na_2SO_4. The crude was finally purified by automated flash chromatography on SiO_2 cartridge (DCM/MeOH, 90:10). The titled compound was obtained by as a yellow solid (0.025 g, 39% Yield). ^{1}**H NMR** (400 MHz, $CDCl_3$) δ 7.38 (s, 1H), 7.27 – 7.23 (m, 1H), 7.06 – 7.01 (m, 2H), 6.80 (s, 1H), 6.73 (d, *J* = 8.5 Hz, 1H), 6.59 (dd, *J* = 8.3, 2.4 Hz, 1H), 6.43 (s, 1H), 6.16 (t, *J* = 2.4 Hz, 1H), 5.98 (dd, *J* = 8.2, 2.3 Hz, 1H), 3.88 (s, 3H), 3.83 (t, *J* = 6.6 Hz, 2H), 3.63 (s, 3H), 3.45 (s, 3H), 3.26 (s, 3H), 1.83 – 1.73 (m, 2H), 1.02 (t, *J* = 7.4 Hz, 3H). ^{13}**C NMR** (101 MHz, $CDCl_3$) δ 160.54, 158.14, 154.79, 152.83, 148.97, 142.36, 130.24, 130.14, 127.99, 126.66, 122.49, 121.43, 110.13, 109.42, 109.22, 108.66, 103.88, 103.50, 99.54, 69.65, 56.07, 55.95, 27.50, 27.32, 22.49, 10.49. **HRMS** (ESI) *m/z* [M + H]+ calcd for $C_{26}H_{29}N_3O_7S$ 528.18045, found 528.18087. **UPLC** retention time: 5.503 min.

3-propoxyphenol (S14)[60].

To a solution of commercially available resorcinol (5.00 g. 45.413 mmol) in DMF (15 mL) K_2CO_3 (3.138 g, 22.707 mmol) and 1-bromopropane (7.214 g, 59.037 mmol) were added. The reaction mixture was stirred at 70°C overnight. The mixture was diluted with H_2O (50 mL), acidified with HCl 2N, and extracted with EtOAc (25 mL x 5). The reunited organic phases were washed with H_2O (30 mL x 6), brine (10 mL x 3), and dried over Na_2SO_4. After removing the solvent via rotavapory evaporation, the crude was purified by automated flash chromatography (PE/EtOAc, 80:20) to obtain **S14** (2.37 g, 34% Yield) as a yellow oil. ^{1}**H NMR** (400 MHz, $CDCl_3$) δ 7.14 (t, *J* = 8.5 Hz, 1H), 6.52 (ddd, *J* = 8.2, 2.2, 1.0 Hz, 1H), 6.48 – 6.40 (m, 2H), 5.21 (bs, 1H), 3.91 (t, *J* = 6.6 Hz, 2H), 1.82 (m, *J* = 7.4 Hz, 2H), 1.05 (t, *J* = 7.4 Hz, 3H). ^{13}**C NMR** (101 MHz, $CDCl_3$) δ 160.41, 156.68, 130.18, 107.80, 107.20, 102.18, 69.69, 22.52, 10.49.

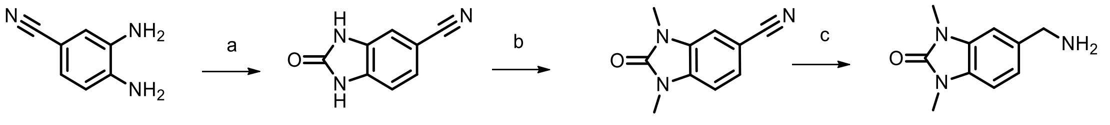

S16 S17 6

Reagents and conditions: (a) 1,1′-carbonyldiimidazole, dry THF, 95 °C, 1.5 h; (b) i. 60% NaH, dry THF, 0 °C-rt, 30 min; ii. CH_3I, dry THF, rt, overnight; (c) Pd/C, P_{H_2} = 6.5 bar, dry EtOH:THF mixture (1:1), rt, overnight.

2-Oxo-2,3-dihydro-1*H*-benzo[*d*]imidazole-5-carbonitrile (S16)[62]

Under N_2 atmosphere, to the solution of 3,4-diaminobenzonitrile (0.200 g, 1.502 mmol) in dry THF (10 mL), *N,N′*-carbonyldiimidazole (0.536 g, 3.305 mmol) was added and the mixture was stirred at 95°C for 1.5 h. Gradually cooled down the reaction mixture to 0 °C, then poured it in iced water (10 mL). Removed the THF by rotary evaporation from the mixture and subsequently acidified with HCl 2N (3 mL).The formed precipitate was subsequently filtered and washed with H_2O (3 x 10 mL) to afford **S16** as a brown powder (0.235 g, 98 % Yield). ^{1}**H NMR** (400 MHz, DMSO-d_6) δ 11.18 (s, 1H), 11.05 (s, 1H), 7.39 (dd, *J* = 8.1, 1.6 Hz, 1H), 7.32 – 7.30 (m, 1H), 7.06 (d, *J* = 8.1 Hz, 1H). ^{13}**C NMR** (101 MHz, DMSO-d_6) δ 155.58, 134.15, 130.39, 126.31, 120.29, 111.82, 109.59, 102.70.

1,3-Dimethyl-2-oxo-2,3-dihydro-1*H*-benzo[*d*]imidazole-5-carbonitrile (S17).[63]

Under N_2 atmosphere, the solution of **S16** (0.308 g, 1.937 mmol) in dry THF (10 mL) was added at 0 °C to a suspension of NaH 60% (0.170 g, 4.426 mmol) in dry THF (5 mL) and stirred for 30 minutes. Iodomethane (0.962 g, 6.778 mmol) was added dropwise and the reaction mixture was stirred at room temperature overnight. The reaction mixture was quenched with EtOAc (10 mL), then diluted with H_2O (10 mL) and extracted with EtOAc (10 mL x 5). The reunited organic phases were washed with brine (10 mL x 2), dried over Na_2SO_4, and evaporated to dryness to give a crude which was purified by automated flash chromatography on SiO_2 cartridge (DCM/MeOH, 98:2) to afford **S17** as a white solid (0.265 g, 73% Yield). **^{1}H NMR** (400 MHz, DMSO-*d*$_6$) δ 7.66 (d, *J* = 1.5 Hz, 1H), 7.52 (dd, *J* = 8.1, 1.5 Hz, 1H), 7.30 (d, *J* = 8.1 Hz, 1H), 3.35 (s, 3H), 3.34 (s, 3H). **^{13}C NMR** (101 MHz, DMSO-*d*$_6$) δ 154.33, 133.76, 130.37, 126.45, 120.24, 11.34, 108.77, 103.00, 27.70, 27.69.

5-(Aminomethyl)-1,3-dimethyl-1*H*-benzo[*d*]imidazol-2(3*H*)-one (6).[63]

In a 12 mL vial, with septum screw cap and a needle, to the solution of **S17** (0.138 g, 0.740 mmol) in a mixture of THF/EtOH 1:1 (9.0 mL) Pd/C 10% (0.070 g) was added. The vial was inserted inside a stainless steel reactor, 3 cycles vacuum/H_2 were performed, and the reaction mixture was left at room temperature under H_2 atmosphere at 6.5 bar pressure overnight. After pressure release, the reaction mixture was filtered over a pad of celite and washed with THF (3 mL x 3). The solvent was evaporated to dryness and the crude was purified by automated flash chromatography on SiO_2 cartridge (DCM/MeOH, 80:20) to afford **6** as a white solid (0.063 g, 44% Yield). **^{1}H NMR** (400 MHz, DMSO-*d*$_6$) δ 7.13 (d, *J* = 0.8 Hz, 1H), 7.08 – 6.98 (m, 2H), 3.73 (s, 2H), 3.31 (s, 3H), 3.30 (s, 3H). **^{13}C NMR** (101 MHz, DMSO-*d*$_6$) δ 154.47, 137.98, 130.11, 128.65, 120.08, 107.54, 106.94, 46.38, 27.41, 27.34. **HRMS** (ESI) *m/z* [M + Na]+ calcd for $C_{10}H_{13}N_3O$ 214.09563, found 214.09545. **UPLC** retention time: 2.586 min.

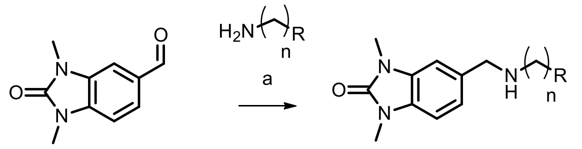

7 n=3; R= $N(CH_3)_2$
8 n= 3; R = $N(CH_2CH_3)_2$
9 n=4; R= $N(CH_3)_2$
10 n=3; R= Pyrrolidin-1yl

Reagents and conditions: (a) Na_2SO_4, $NaBH_4$, DCM then MeOH, rt, overnight.

General Procedure A for reductive amination

Under N_2 atmosphere, to the solution of commercially available 1,3-dimethyl-2-oxo-2,3-dihydro-1*H*-benzo[*d*]imidazole-5-carbaldehyde (1.0 equiv) in dry DCM, Na_2SO_4 (catalytic amount) and the appropriate amine (1.2 equiv) were added. The reaction mixture was stirred at room temperature for 18 h. Then, the reaction mixture was filtered and the filtrate evaporated to dryness. The solid was dissolved in dry MeOH and at 0 °C $NaBH_4$ (2.0 equiv) was added. The mixture was then stirred at room temperature 18 h. The solvent was then evaporated *via* rotary evaporation by carefully avoiding light exposure and overheating and the crude was directly purified under dark as described below.

5-(((3-(Dimethylamino)propyl)amino)methyl)-1,3-dimethyl-1*H*-benzo[*d*]imidazol-2(3*H*)-one (7).

The titled compound was obtained by following representative procedure **A** using as reactant *N$_1$,N$_1$*-dimethylpropane-1,3-diamine (0.045 g, 0.441 mmol) and after purification by flash chromatography on SiO_2 (DCM/MeOH 9:1 then DCM/MeOH/Et_3N 90:8:2) as a light yellow oil (0.055 g, 54% Yield). **^{1}H NMR** (400 MHz, $CDCl_3$) δ 7.03 (dd, *J* = 7.9, 1.5 Hz, 1H), 6.99 (d, *J* = 1.5 Hz, 1H), 6.89 (d, *J* = 7.9 Hz, 1H), 3.83 (s, 2H), 3.42 – 3.39 (m, 6H), 2.69 (t, *J* = 7.1 Hz, 2H), 2.36 – 2.29 (m, 2H), 2.21 (s, 6H), 1.69 (p, *J* = 7.2 Hz, 2H). **^{13}C NMR** (101 MHz, $CDCl_3$) δ 154.84, 133.86, 130.23, 129.09, 121.13, 107.31, 106.98, 58.10, 54.31, 47.94, 45.58 (2C), 28.03, 27.20 (2C). **HRMS** (ESI) *m/z* [M + H]+ calcd for $C_{15}H_{24}N_4O$ 277.20284, found 277.20252. **UPLC** retention time: 2.451 min.

5-(((3-(Diethylamino)propyl)amino)methyl)-1,3-dimethyl-1*H*-benzo[*d*]imidazol-2(3*H*)-one (8).
The titled compound was obtained by following general procedure **A** using as reactant *N1,N1*-diethylpropane-1,3-diamine (0.057 g, 0.441 mmol) and after purification by flash chromatography on SiO_2 (DCM/MeOH 9:1 then DCM/MeOH/Et_3N 90:8:2) as a light yellow oil (0.038 g, 34% yield). **^{1}H NMR** (400 MHz, $CDCl_3$) δ 7.02 (dd, *J* = 7.9, 1.1 Hz, 1H), 6.99 (s, 1H), 6.88 (d, *J* = 7.9 Hz, 1H), 3.81 (s, 2H), 3.41 – 3.37 (m, 6H), 2.68 (t, *J* = 6.9 Hz, 2H), 2.54 – 2.45 (m, 6H), 1.73 – 1.63 (m, 2H), 0.99 (t, *J* = 7.1 Hz, 6H). **^{13}C NMR** (101 MHz, $CDCl_3$) δ 154.84, 133.68, 130.24, 129.11, 121.17, 107.36, 106.97, 54.26, 51.35, 48.34, 46.84 (2C), 27.21, 27.20, 27.05, 11.64 (2C). **HRMS** (ESI) *m*/*z* [M + H]+ calcd for $C_{17}H_{28}N_4O$ 305.23414, found 305.23379. **UPLC** retention time: 1.332 min.

5-(((4-(Dimethylamino)butyl)amino)methyl)-1,3-dimethyl-1*H*-benzo[*d*]imidazol-2(3*H*)-one (9).
The titled compound was obtained by following general procedure **A** using as reactant *N1,N1*-dimethylbutane-1,4-diamine (0.051 g, 0.441 mmol) and after purification by flash chromatography on SiO_2 (DCM/MeOH 9:1 then DCM/MeOH/Et_3N 90:8:2) as a light yellow oil (0.039 g, 36% yield). **^{1}H NMR** (400 MHz, $CDCl_3$) δ 7.11 (s, 1H), 7.05 (dd, *J* = 7.9, 1.4 Hz, 1H), 6.90 (d, *J* = 7.9 Hz, 1H), 3.87 (s, 2H), 3.44 – 3.38 (m, 6H), 2.71 (t, *J* = 6.7 Hz, 2H), 2.30 (t, *J* = 7.0 Hz, 2H), 2.18 (s, 6H), 1.69 – 1.60 (m, 2H), 1.60 – 1.50 (m, 2H). **^{13}C NMR** (101 MHz, $CDCl_3$) δ 154.83, 133.05, 130.29, 129.25, 121.25, 107.51, 106.98, 59.51, 53.99, 49.19, 45.22 (2C), 27.81, 27.23, 27.22, 25.56. **HRMS** (ESI) *m*/*z* [M + H]+ calcd for $C_{16}H_{26}N_4O$ 291.21849, found 291.21785. **UPLC** retention time: 1.306 min.

1,3-Dimethyl-5-(((3-(pyrrolidin-1-yl)propyl)amino)methyl)-1*H*-benzo[*d*]imidazol-2(3*H*)-one (10).
The titled compound was obtained by following representative procedure **A** using as reactant 3-(pyrrolidin-1-yl)propan-1-amine (0.040 g, 0.315 mmol) and after purification by flash chromatography on SiO_2 (DCM/MeOH 9:1 then DCM/MeOH/Et_3N from 90:9:1) as a light yellow oil (0.061 g, 76% yield). **^{1}H NMR** (400 MHz, $CDCl_3$) δ 7.03 – 6.99 (m, 1H), 6.97 (s, 1H), 6.88 (d, *J* = 7.9 Hz, 1H), 3.82 (s, 2H), 3.41 – 3.36 (m, 6H), 2.70 (t, *J* = 7.0 Hz, 2H), 2.54 – 2.46 (m, 6H), 1.79 – 1.70 (m, 6H). ^{13}C NMR (101 MHz, $CDCl_3$) δ 154.83, 133.68, 130.24, 129.12, 121.13, 107.32, 106.98, 54.83, 54.26(2C), 54.17, 48.09(2C), 29.05, 27.21, 23.43(2C).
HRMS (ESI) *m*/*z* [M + H]+ calcd for $C_{17}H_{26}N_4O$ 303.21849, found 303.21808. **UPLC** retention time: 2.319 min.

2.3 Crystallographic studies

TRIM33*α* PHD-BRD expression and purification

The gene coding sequence for TRIM33*α* (residues 882-1087), cloned in the pET28a(+) vector (including a TEV-cleavable His_6-tag) within the NdeI – XhoI restriction sites, was purchased from GenScript (Piscataway, NJ). The expression plasmid was introduced by thermal shock in the BL21(DE3) *E. coli* strain. Bacterial cells were grown in Luria-Bertani medium supplemented with 50 mg L^{-1} kanamycin at 37 °C. Over-expression levels of the protein was obtained by 16 h of induction at 20 °C in the presence of 0.25 mM isopropyl β-D-1-thiogalactopyranoside (IPTG) and 0.1 mM zinc chloride when the OD_{600nm} reached values between 0.6 – 0.8. Cells, harvested by centrifugation, were resuspended in buffer A (500 mM NaCl and 20 mM TRIS, pH 8) containing 20 mM imidazole and lysozyme (0.5 mg mL^{-1}) and disrupted by sonication after 1 h incubation on ice. The soluble cellular fraction, separated by centrifugation (13500 x g, 1 h, 4 °C), was purified by nickel-affinity chromatography (HisTrap FF 5 mL column, GE Healthcare) following the manufacturer's instructions. Protein elution was achieved when the imidazole concentration reached 250 mM. Fractions containing the target protein, identified by SDS-PAGE analysis, were pooled, and extensively dialyzed in buffer A. His_6-tag cleavage was performed during the dialysis by means of in-house produced His_6-tagged TEV protease (HT-TEV) directly added inside the membrane (0.05 mg per mg of target protein)[64]. After 24 h, the tag cleavage was almost complete (> 98 %, verified by SDS-PAGE analysis), and the mature protein was subjected to a second nickel affinity chromatography. The effective tag removal was confirmed by western blot analysis using an HRP-conjugated His-tag monoclonal antibody (Thermo Fisher Scientific). The purity of the mature protein was estimated as > 98% by SDS-PAGE and MALDI-TOF mass spectrometry analyses. The final yield obtained after protein purification was 30 mg L^{-1} bacterial culture for TRIM33*α* PHD-BRD.

Crystallization

Before crystallization experiments, TRIM33 α PHD-BRD was concentrated to 15 mg mL^{-1} in buffer B (100 mM NaCl and 20 mM TRIS, pH 8) added by 5 mM dithiothreitol. TRIM33α PHD-BRD was crystallized as reported by Xi et al.[39], with minor modifications. Briefly, crystals of the protein were grown using the hanging and sitting drop vapour diffusion method[65] by mixing equal volumes of protein and precipitant (0.2 M calcium chloride and 20 % wt/vol PEG-3350) solution, equilibrated over a 500 µL reservoir at 8 °C. Crystals grew within 24 hours and reached their maximum size by a week. The complexes of TRIM33α PHD-BRD with 8, 9 and 10 were obtained by the soaking technique. Preformed protein crystals were soaked for 30 minutes with 10 mM of each compound (solubilized in DMSO, without exceeding a DMSO/crystal solution ratio of 1:9). Crystals of the complexes were transferred to the cryoprotectant solution (obtained by adding 20 % v/v ethylene glycol to the precipitant solutions) and then flash frozen in liquid nitrogen.

Data collection, structure solution and refinement

Diffraction data were collected at 100 K using synchrotron radiation at the Diamond Light Source (DLS, Didcot, UK) beamline I04, equipped with an Eiger2 XE 16M detector. Data were integrated using XDS[66] and scaled with SCALA[67,68] from the CCP4 suite[69]. Crystals of TRIM33α PHD-BRD in complex with 8, 9, and 10 belonged to the hexagonal space group $P6_522$. Molecular replacement was performed using the software Molrep[70] and the structure of TRIM33α PHD-BRD (PDB id 3u5m[39], excluding non-protein atoms and water molecules) as searching model. All structures were refined through the program REFMAC5[71] from CCP4 suite. The molecular graphic software Coot[72] was used for manual rebuilding and modelling of missing atoms in the electron density and to add solvent molecules. In each structure, the inspection of the Fourier difference map clearly evidenced the presence of the ligand inside the KAc binding site that was modelled accordingly. The final models were inspected manually and checked with Coot and PROCHECK[73] and then validated through the Protein Data Bank (PDB) deposition tools. Structural figures were generated using the molecular graphic software CCP4 mg[74]. Data collection, processing, and refinement statistics are summarized in Table 5. Final coordinates and structure factors were deposited in the Protein Data Bank (PDB) under the codes 8BD8 (TRIM33α PHD-BRD – 8), 8BDY (TRIM33α PHD-BRD – 9), and 8BD9 (TRIM33α PHD-BRD – 10).

Table S6. Data collection and processing and refinement statistics. Values for the outer shell are given in parentheses.

	**TRIM33α PHD-BRD compound 8**	**TRIM33α PHD-BRD compound 9**	**TRIM33α PHD-BRD compound 10**
PDB ID codes	**8BD8**	**8BDY**	**8BD9**
DATA COLLECTION STATISTICS			
Diffraction source	I04 (DLS)	I04 (DLS)	I04 (DLS)
Wavelength (Å)	0.9795	0.9795	0.9795
Temperature (K)	100	100	100
Detector	Eiger2 XE 16M	Eiger2 XE 16M	Eiger2 XE 16M
Crystal–detector distance (mm)	329.4	366.6	329.4
Exposure time per image (s)	0.1	0.005	0.1
Space group	$P6_522$	$P6_522$	$P6_522$
No. of subunits in ASU	1	1	1
$a = b$, c (Å)	80.01, 134.47	79.96, 135.50	79.95, 134.95
Resolution range (Å)	134.47–3.10 (3.27–3.10)	135.50-3.05 (3.21-3.05)	134.95– 3.20 (3.37– 3.20)
Total no. of reflections	38302 (5842)	72965 (11190)	17680 (2603)
No. of unique reflections	4853 (692)	5355 (761)	4598 (633)
Completeness (%)	97.6 (99.4)	100.0 (100.0)	99.5 (99.8)
Redundancy	7.9 (8.4)	13.6 (14.7)	3.8 (4.1)
$\langle I/\sigma(I)\rangle$	11.0 (2.3)	10.0 (2.2)	11.2 (2.3)
R_{meas}	0.171 (1.186)	0.189 (1.118)	0.077 (0.625)
Overall B factor from Wilson plot (Å^2)	64.5	69.0	100.3

REFINEMENTS STATISTICS			
Resolution range (Å)	69.30-3.10 (3.18-3.10)	69.25-3.05 (3.13-3.05)	61.68-3.20 (3.28-3.20)
Completeness (%)	96.4 (99.2)	99.98 (100.0)	99.3 (99.7)
No. of reflections, working set	4645 (335)	5062 (363)	4307 (291)
No. of reflections, test set	207 (16)	256 (23)	256 (21)
Final R_{cryst}	0.2102 (0.293)	0.2287 (0.345)	0.2319 (0.359)
Final R_{free}	0.3383 (0.547)	0.3342 (0.511)	0.3485 (0.442)
**No. of non-H atoms**			
Protein	1471	1425	1384
Inhibitor	12	12	12
Water	19	20	5
Zn ions	2	2	2
Calcium ions	1	1	1
Total	1505	1460	1404
R.m.s. deviations Bonds (Å)	0.005	0.008	0.005
Angles (°)	1.679	1.771	1.413
Average *B* factors (Å^2)	60.5	69.3	86.8
Estimate error on coordinates based on R value (Å)	0.615	0.559	0.652
**Ramachandran plot**			
Most favored (%)	82.1%	85.9%	83.5%
Allowed (%)	17.9%	14.1%	16.5%
RSCC Inhibitor	0.91	0.93	0.87

Bibliography

36. Sekirnik, A.R.; Reynolds, J.K.; See, L.; Bluck, J.P.; Scorah, A.R.; Tallant, C.; Lee, B.; Leszczynska, K.B.; Grimley, R.L.; Storer, R.I.; et al. Identification of Histone Peptide Binding Specificity and Small- Molecule Ligands for the TRIM33α and TRIM33β Bromodomains Published. *ACS Chem. Biol.* **2022**, *17*, 2753–2768. https://doi.org/10.1021/acschembio.2c00266.
39. Xi, Q.; Wang, Z.; Zaromytidou, A.-I.; Zhang, X.H.-F.; Chow-Tsang, L.-F.; Liu, J.X.; Kim, H.; Barlas, A.; Manova-Todorova, K.; Kaartinen, V. A Poised Chromatin Platform for TGF-β Access to Master Regulators. *Cell* **2011**, *147*, 1511–1524. https://doi.org/10.1016/j.cell.2011.11.032.
42. Palmer, W.S.; Poncet-montange, G.; Liu, G.; Petrocchi, A.; Reyna, N.; Subramanian, G.; Thero, J.; Yau, A.; Kost-alimova, M.; Bardenhagen, J.P.; et al. Structure-Guided Design of IACS-9571, a Selective High-A Ffi Nity Dual TRIM24-BRPF1 Bromodomain Inhibitor. *J. Med. Chem.* **2015**, *59*, 1440–1454. https://doi.org/10.1021/acs.jmedchem.5b004055.
57. Tsai, W.-W.; Wang, Z.; Yiu, T.T.; Akdemir, K.C.; Xia, W.; Winter, S.; Tsai, C.-Y.; Shi, X.; Schwarzer, D.; Plunkett, W. TRIM24 Links a Non-Canonical Histone Signature to Breast Cancer. *Nature* **2010**, *468*, 927–932. https://doi.org/10.1038/nature09542.
60. Zeng, L.; Yap, K.L.; Ivanov, A. V; Wang, X.; Mujtaba, S.; Plotnikova, O.; Rauscher III, F.J.; Zhou, M.-M. Structural Insights into Human KAP1 PHD Finger–Bromodomain and Its Role in Gene Silencing. *Nat. Struct. Mol. Biol.* **2008**, *15*, 626–633, doi:10.1038/nsmb.1416.
61. Burdick, D.J.; Liang, J. Preparation of Pyrimidines as Aurora Kinase Inhibitors. Patent WO2007120339, 2007 October 25.
62. Steeneck, C.; Gege, C.; Richter, F.; Hochguertel, M.; Feuerstein, T.; Bluhm, H. Preparation of Amide Containing Heterobicyclic Metalloprotease Inhibitors. Patent WO2006128184, 2007 March 08.
63. Stazi, G.; Battistelli, C.; Piano, V.; Mazzone, R.; Marrocco, B.; Marchese, S.; Louie, S.M.; Zwergel, C.; Antonini, L.; Patsilinakos, A.; et al. Development of Alkyl Glycerone Phosphate Synthase Inhibitors: Structure-Activity Relationship and Effects on Ether Lipids and Epithelial-Mesenchymal Transition in Cancer Cells. *Eur. J. Med. Chem.* **2019**, *163*, 722–735, doi:10.1016/j.ejmech.2018.11.050.
64. Kapust, R.B.; Tözsér, J.; Fox, J.D.; Anderson, D.E.; Cherry, S.; Copeland, T.D.; Waugh, D.S. Tobacco Etch Virus Protease: Mechanism of Autolysis and Rational Design of Stable Mutants with Wild-Type Catalytic Proficiency. *Protein Eng.* **2001**, *14*, 993–1000.
65. Benvenuti, M.; Mangani, S. Crystallization of Soluble Proteins in Vapor Diffusion for X-Ray Crystallography. *Nat. Protoc.* **2007**, *2*, 1633–1651.
66. Kabsch, W. Xds. *Acta Crystallogr. Sect. D Biol. Crystallogr.* **2010**, *66*, 125–132.
67. Evans, P.R. An Introduction to Data Reduction: Space-Group Determination, Scaling and Intensity Statistics. *Acta Crystallogr. Sect. D Biol. Crystallogr.* **2011**, *67*, 282–292, doi:10.1107/S090744491003982X.
68. Evans, P. Scaling and Assessment of Data Quality. *Acta Crystallogr. Sect. D Biol. Crystallogr.* **2006**, *62*, 72–82, doi:10.1107/S0907444905036693.
69. Winn, M.D.; Ballard, C.C.; Cowtan, K.D.; Dodson, E.J.; Emsley, P.; Evans, P.R.; Keegan, R.M.; Krissinel, E.B.; Leslie, A.G.W.; McCoy, A.; et al. Overview of the CCP4 Suite and Current Developments. *Acta Crystallogr. Sect. D Biol. Crystallogr.* **2011**, *67*, 235–242, doi:10.1107/S0907444910045749.
70. Vagin, A.; Teplyakov, A. Molecular Replacement with MOLREP. *Acta Crystallogr. Sect. D Biol. Crystallogr.* **2010**, *66*, 22–25.
71. Murshudov, G.N.; Skubák, P.; Lebedev, A.A.; Pannu, N.S.; Steiner, R.A.; Nicholls, R.A.; Winn, M.D.; Long, F.; Vagin, A.A. REFMAC5 for the Refinement of Macromolecular Crystal Structures. *Acta Crystallogr. Sect. D Biol. Crystallogr.* **2011**, *67*, 355–367.
72. Emsley, P.; Lohkamp, B.; Scott, W.G.; Cowtan, K. Features and Development of Coot. *Acta Crystallographica Section D: Biological Crystallography* **2010**, 66, 486-501.
73. Laskowski, R.A.; MacArthur, M.W.; Moss, D.S.; Thornton, J.M. IUCr. *PROCHECK: a program to check the stereochemical quality of protein structures. J. app. Crystallogr.* **1993**, 26, 283-291.
74. Potterton, E.; McNicholas, S.; Krissinel, E.; Cowtan, K.; Noble, M. The CCP4 Molecular-Graphics Project. *Acta Crystallogr. Sect. D Biol. Crystallogr.* **2002**, *58*, 1955–1957.
